# A systematic review of factors affecting wildlife survival during rehabilitation and release

**DOI:** 10.1371/journal.pone.0265514

**Published:** 2022-03-17

**Authors:** Holly R. Cope, Clare McArthur, Christopher R. Dickman, Thomas M. Newsome, Rachael Gray, Catherine A. Herbert

**Affiliations:** 1 Sydney School of Veterinary Science, Faculty of Science, University of Sydney, NSW, Australia; 2 School of Life and Environmental Sciences, Faculty of Science, University of Sydney, NSW, Australia; Auburn University, UNITED STATES

## Abstract

Millions of native animals around the world are rescued and rehabilitated each year by wildlife rehabilitators. Triage and rehabilitation protocols need to be robust and evidence-based, with outcomes consistently recorded, to promote animal welfare and better understand predictors of wildlife survival. We conducted a global systematic review and meta-analysis of 112 articles that reported survival rates of native mammals and birds during rehabilitation and after release to determine intrinsic and extrinsic factors associated with their survival. We assessed survival during rehabilitation and in the short- and long-term post-release, with the hypothesis that survival will vary as a function of species body size, diel activity pattern, trophic level and study location (region of the world). We aimed to determine the direction of effect of these factors on survival to assist in decision-making during triage and rehabilitation. Results showed that mammals and birds were equally likely to survive all stages of rehabilitation, and survival rates varied between locations. Birds in North America had the poorest survival rates post-release, particularly long-term, as did diurnal and carnivorous birds in the short-term post-release. Anthropogenic factors such as motor vehicle collisions and domestic or feral animal attack contributed to morbidity and post-release mortality in 45% (168 of 369) of instances. The reasons for rescue and associated severity of diagnosis were commonly reported to affect the likelihood of survival to release, but factors affecting survival were often species-specific, including bodyweight, age, and characteristics of the release location. Therefore, evidence-based, species-specific, and context-specific protocols need to be developed to ensure wildlife survival is maximised during rehabilitation and post-release. Such protocols are critical for enabling rapid, efficient rescue programs for wildlife following natural disasters and extreme weather events which are escalating globally, in part due to climate change.

## Introduction

Wildlife rehabilitation is practiced in many countries, resulting in the rescue, care, and release of millions of animals every year [1]. Wildlife commonly require rehabilitation due to collisions with motor vehicles, abandonment and domestic animal attack (among others), while many targeted rescues are in response to environmental disasters such as oil spills or wildfire [2, 3]. Wildlife rehabilitation has been defined as “the act of providing temporary care for injured, sick or orphaned wildlife with the goal of releasing them back into the wild” [4]. Although there is limited evidence of the fate and contribution of released animals to the conservation of populations or species [1, 5], there are other reasons why rehabilitation can be valuable, or valued. For example, release of rehabilitated animals may help to supplement and maintain existing populations [1, 3, 6–8]. Wildlife care and rehabilitation often garner attention from the community and media and serve as effective education and fundraising tools [2, 9]. The knowledge and experience gained while rehabilitating commonly encountered species can also support the care of threatened species [2]. Rehabilitation can also be supported for ethical, legal and welfare concerns in certain situations, varying by country [10, 11]. For these reasons, wildlife rehabilitation is likely to continue, and assessments of the factors affecting wildlife survival during rehabilitation and release can help to inform future directions.

To identify factors influencing the success of wildlife rehabilitation, it is first necessary to define “success”, and this may vary among stakeholders. From a wildlife rehabilitator’s perspective, success could constitute recovery from initial injuries and release back into the wild [12]. Success for the individual animal could entail recovery from injuries and long-term survival in the wild with successful reproduction [13]. Success at the population level could constitute persistence of populations where rehabilitated animals are released, with released animals maintaining individual territories and contributing to the reproductive population, without introducing deleterious genetic alleles or disease, or pushing the population beyond the carrying capacity of the habitat or exacerbating intraspecific competition [6, 7]. Such measures of success at the population level likely mirror those for the success of a conservation reintroduction, which has been defined as the creation of a self-sustaining population [14]. In this review, we consider success in terms of individual animal survival during care, and short- and long-term survival post-release. However, the potential impact of released rehabilitated animals at the population level remains a knowledge gap for many species.

Communities and native animals rely on volunteer wildlife rehabilitators to rescue, rehabilitate and release injured or orphaned wildlife [5, 15, 16]. However, few studies have used an experimental approach to assess rehabilitation methods or factors associated with survival to release. Consequently, rehabilitators rely largely on an evolution of methods through trial and error, shared knowledge and guidelines developed by wildlife authorities [1] (see examples [17, 18]). For example, habituation to humans can reduce survival in the wild if animals do not display appropriate predator avoidance behaviours [6, 19]. However, few studies have quantified the effect of different measures employed by rehabilitators to avoid habituation [5]. Similarly, limited numbers of studies have monitored survival outcomes post-release in relation to the rehabilitation methods used [19]. Given the likelihood of increased frequency and severity of natural disasters in the future, including wildfires [20, 21], combined with increased threats of urbanisation such as motor vehicle collisions, dog attack and entanglement in netting or wire [16], it is likely that wildlife rescue, rehabilitation and release will play an increasingly important role in conservation efforts over time [6, 22, 23]. As such, it is valuable to assess current survival rates and factors associated with the success of rehabilitation of rescued wildlife.

We used a systematic approach to review survival rates of native mammals and birds during rehabilitation and post-release to determine factors associated with survival. We focused on mammals and birds as these classes are commonly rescued and rehabilitated, with survival data subsequently reported in the literature. The effects of a range of intrinsic and extrinsic factors on survival were evaluated to develop a framework of key considerations for wildlife rehabilitation, and to guide future research on best-practice rehabilitation methods. Specifically, we hypothesised that survival rates during rehabilitation and post-release will vary as a function of species traits that could impact susceptibility to anthropogenic and environmental threats, such as body size, trophic level and diel activity pattern, and survival will vary between regions of the world. Thus, survival likelihood will be species- and context-specific.

## Methods

### Systematic review scope

A standard systematic search strategy, as outlined by Pullin and Stewart [24], was used to identify peer-reviewed and grey literature reporting mammal and bird survival during wildlife rehabilitation around the world. Search results were recorded using a Preferred Reporting Items for Systematic Reviews and Meta-Analyses (PRISMA) flow diagram [25]. Online databases Scopus and Web of Science were searched along with relevant conference proceedings, reference lists of selected articles (backwards search) and Google Scholar. The literature search was completed by H. Cope over a three-month period from January to March 2021, and included 50 journals, three of which were particularly relevant–*Animal Welfare*, *Journal of Raptor Research*, and *Journal of Wildlife Rehabilitation*. Additionally, 16 relevant books, symposium proceedings, reports and theses were used. We used search terms relating to the location of the study, focal taxa, rehabilitation intervention and survival, without date limits (S1 Table). The online systematic review tool, SysRev, (sysrev.com) was used to reduce the returned articles based on title, abstract and keywords. As thousands of articles were returned, they were sorted by relevance then searched until there were 50 consecutive non-selected articles (an arbitrary number, usually representing two pages of search results, past which relevant articles were unlikely to be found). Full texts were then reviewed against selection criteria as follows: research was conducted on native mammals or birds that entered care for any reason; the sample size and a survival measure and timeframe (e.g. number of mortalities, annual survival rate, minimum known alive) during rehabilitation or post-release were reported; and the article (or abstract) was available online and in English. Few studies included a control group or intervention other than rehabilitation, so this was not a requirement for inclusion. Where studies included a control group, this involved monitoring a sample of wild counterparts, uninjured animals, or known baseline survival rates for the resident population.

## Statistical analyses

### Definition of variables

One reviewer created a summary of study characteristics for each article detailing study species, sample size, study location, reason for entry into care, percentage of unassisted deaths in care (i.e. excluding euthanasia), percentage survival to release from care, short- and long-term survival post-release, factors reported as affecting survival, and causes of mortality. Post-release survival was categorised as short-term (< six months) or long-term (> six months) to minimise bias between study outcomes. These time frames were selected because some studies reported survival at multiple time points, and monitoring varied from 14 days to six years. We considered six months to be a reasonable time for animals to settle into their environment and forage independently in more than one season. Location was also included in the analyses to disentangle the potential effects of the suite of species found at a location, varying rehabilitation policies and practices around the world, or other abiotic processes from the biological characteristics of species.

We used the Encyclopedia of Life (https://eol.org/) to categorise species according to class (Aves, Mammalia), diel activity pattern (any time, crepuscular, diurnal, nocturnal), average adult weight (small < 5.5 kg, medium 5.5–100 kg, large > 100 kg) and trophic level (herbivores [primary consumers], omnivores and carnivores/pescatarians [secondary and tertiary consumers], apex carnivores). The adult weight classes were designed to separate species according to the Australian critical weight range, i.e. 35 g– 5.5 kg [26], from other species within the same order and trophic level as we expected smaller species to be more susceptible to predation and mortality. The largest weight class encompasses mammals that have a greater probability of being threatened than the medium weight class [27]. There were no avian species in the largest weight class. Trophic levels were designed to separate dietary niches such as predators from prey species. These sub-groups were considered sufficient to reduce risk of bias from individual studies. There were insufficient samples to further stratify studies based on the methods used.

The reasons for entry into care and causes of post-release mortality were grouped into three categories—anthropogenic, environmental, and non-specific (those that could not be attributed)—and reported as frequencies. Factors affecting survival during rehabilitation or after release were categorised as being related to the event that precipitated entry into care (e.g. severity and type of injury), intrinsic or individual traits (e.g. body size, behaviour and age), intervention (e.g. rescue protocols, choice of diet and pre-release training), release environment (e.g. timing of release, release method and habitat quality), and human-wildlife interface (e.g. hunting activity and urban expansion), and summarised. These factors were shown statistically or observationally to affect survival in the reviewed articles.

#### Statistical methods

Statistical analyses were performed in R (version 4.0.5) [28]. A mixed-effects meta-regression model in the METAFOR package was used to assess the relationship between survival and characteristics of the study species. Species was included as a random effect to account for multiple studies on the same species. Effect sizes were weighted by the sample size due to a lack of reported error measures for most articles (survival was generally reported as percentage known alive), based on the expectation that variance will decrease with larger sample sizes. Survival rates and sample sizes were then used to calculate log-odds of survival. Survival was initially compared between Aves and Mammalia for each stage of rehabilitation, being the unassisted death rate in care (i.e. deaths not resulting from euthanasia), survival to the end of rehabilitation (i.e. release to the wild or long-term captivity), short-term survival post-release, and long-term survival post-release. Each class was then assessed separately to determine the effect of factors hypothesised to affect survival including study location (Oceania [and Asia], North America, Europe, Africa and Others [Middle East, Southern America]) and species’ diel activity pattern, adult weight class and trophic level at each stage of rehabilitation. Strength of association was first assessed between all paired combinations of predictors using a Fisher’s exact test, and predictors with a relationship (p < 0.05) were not included together in models. All combinations of predictors were modelled and Akaike’s Information Criterion (AIC) [29] was used to select the best model with the lowest AIC value by ≥ 2 points. Where no model satisfied this criterion, the most parsimonious model (least number of predictors) within two points of the lowest AIC value was selected. Between-study heterogeneity was reported as *I*^*2*^ [30]. Probabilities of survival were calculated as a back-transformation of log-odds for single predictor models for simplicity of interpretation.

Publication bias can exist where small studies with small effect sizes are not published or there is selective reporting within studies. We tested for bias in METAFOR by creating a funnel plot of effect size versus sampling variance of the effect size for each survival measure [31]. Egger’s test was used for funnel plot asymmetry and trim-and-fill analysis [29] was used to estimate magnitude of publication bias.

## Results

**Fig 1:**
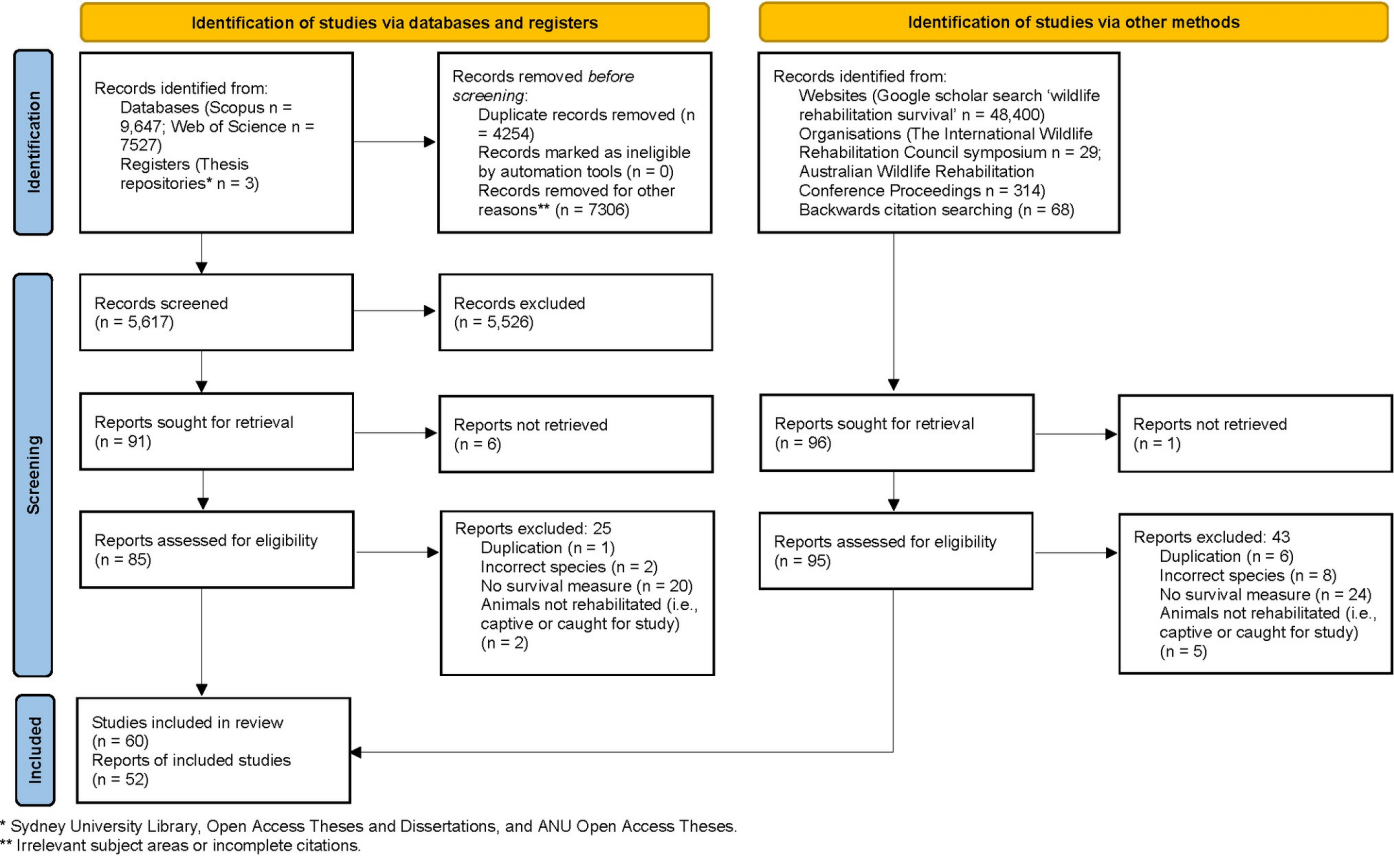
PRISMA flow diagram of systematic search strategy and results. Adapted from Page et al. [25].

The literature search yielded 5617 publications, of which 187 were initially selected; after reviewing the full texts, 112 articles satisfied all inclusion criteria (Fig 1). Several articles presented independent survival results for more than one species or population, and these results were analysed separately and hereafter referred to as studies, totalling 125. Articles were published between 1981 and 2021. Sample sizes ranged from 2–22,344 (mean 1076, median 63). Eighteen articles included a control group with sample sizes ranging from 3–5726 (mean 684, median 23). Retrospective studies using wildlife rescue databases contributed to the large mean sample sizes. Research was mostly conducted in Australia, Europe, North America and Southern Africa (Table 1). Funnel plot analysis showed an estimated lack of 13 studies with large effect sizes for unassisted death in care (p = 0.0006) resulting in possible underestimation in our results, six missing studies with small effect sizes for both rehabilitation survival (p = 0.0038) and post-release short-term survival (p = 0.0328) resulting in possible overestimation, and no publication bias in long-term post-release survival (p = 0.1397; S2 Table).

**Table 1:** Number of published articles from each country or region reporting survival of wildlife during rehabilitation, post-release or during both phases.

Region	Rehabilitation	Post-release	Both	Total*
Oceania (and Asia)				
Australia	7	11	6	24 (21.4)
East Asia	1	0	0	1 (0.9)
New Zealand	1	0	0	1 (0.9)
South Asia	0	1	1	2 (1.8)
North America				
Canada	2	0	0	2 (1.8)
North America	10	14	5	29 (25.9)
Several northern hemisphere countries	0	1	0	1 (0.9)
Europe	15	14	3	32 (28.6)
Africa				
Southern Africa	6	6	5	17 (15.2)
Others				
Middle East	2	0	0	2 (1.8)
South America	0	1	0	1 (0.9)
**Grand total**	44	48	19	112 (100)

* Values presented as total (percentage of grand total).

### Reason for entry into care and causes of mortality

Most reasons for entry into care were anthropogenic in origin, followed by non-specific causes, with a small proportion attributed to natural environmental causes (Table 2). Most mortalities in care occurred as a direct result of the initial reason for admission, either by euthanasia or unassisted death. In eight articles, secondary complications caused death, as sequelae of the initial reasons for admission or resulting from rescue procedures, treatment, or captivity. The mean unassisted death rate after entry into care was 17.9% (2.4) and 18.2% (4.0) for birds and mammals, respectively (overall 17.9% (2.1); presented as mean (SE)). The most common known causes of post-release mortality were predation (by domestic, feral, native, and conspecific predators, equalling 24% of all causes), vehicle collision, and illegal shooting or legal hunting.

**Table 2:** Frequency of studies reporting various anthropogenic, environmental and non-specific causes for entry of wildlife into care, and mortality post-release.

Origin of cause for rehabilitation or mortality	Cause of entry into care*	Cause of mortality post-release*
**Anthropogenic**	**117 (48.0)**	**51 (40.8)**
Collision–motor vehicle	13 (5.3)	16 (12.8)
Gunshot or poaching	8 (3.3)	15 (12.0)
Domestic or feral animal attack or predation	11 (4.5)	11 (8.8)
Oil spill	21 (8.6)	0 (0)
Toxicosis or poisoning	12 (4.9)	1 (0.8)
Electrocution/collision with powerlines	8 (3.3)	3 (2.4)
Collision–structure	10 (4.1)	0 (0)
Confiscated	10 (4.1)	N/A
Relocated or displaced	4 (1.6)	1 (0.8)
Entanglement	3 (1.2)	1 (0.8)
GPS or VHF collar injury	N/A	1 (0.8)
Other (e.g. capture myopathy, trap, tree felling, human interference)	17 (7.0)	2 (1.6)
**Environmental**	**24 (9.8)**	**36 (28.8)**
Disease	19 (7.8)	6 (4.8)
Predation by native predator or conspecific	1 (0.4)	19 (15.2)
Misadventure (burrow collapse, drowning, ingested wasp, killed by elephant)	0 (0)	6 (4.8)
Fire, flood or storm	3 (1.2)	2 (1.6)
Natural mortality–age-related	N/A	3 (2.4)
Heat stress	1 (0.4)	0 (0)
**Non-specific**	**103 (42.2)**	**38 (30.4)**
Generic trauma or unidentified illness (i.e. Sick, injured, trauma, exhaustion)	41 (16.8)	7 (5.6)
Orphaned or stranded juvenile	46 (18.9)	N/A
Malnutrition	8 (3.3)	5 (4.0)
Unresolved initial ailment	N/A	7 (5.6)
Unknown causes	8 (3.3)	19 (15.2)
Studies that did not cite a specific reason	10	11

* Values presented as number of studies, not number of individuals, with percentage of the total in brackets.

### Factors associated with survival

**Fig 2:**
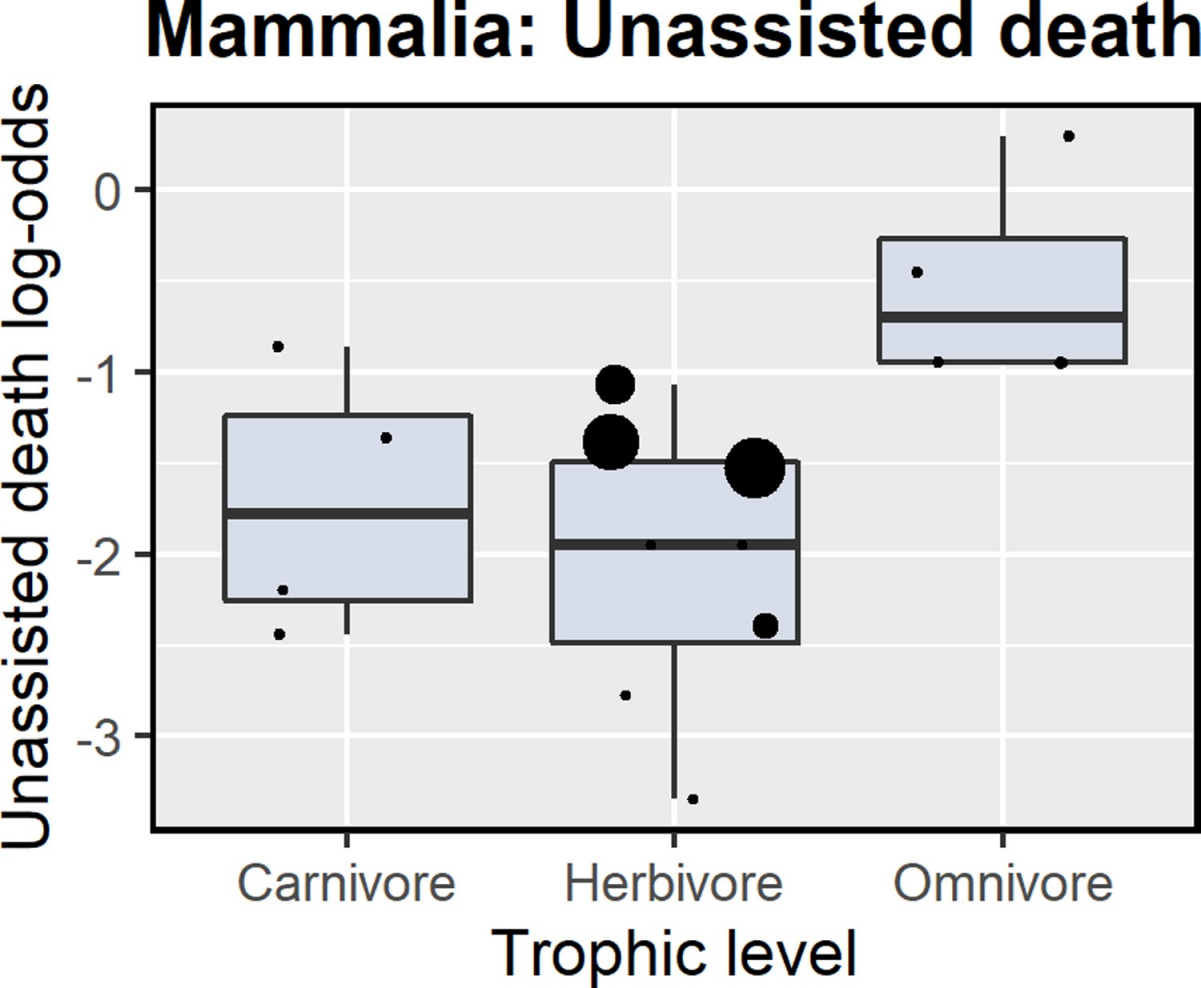
Distribution of log-odds across factors affecting unassisted death rates for mammalian species. Note that each point represents the log-odds of survival of one study-species combination, and the size of the point is proportional to the inverse variance of the log-odds (i.e. larger points have more weight).

There were no differences between classes for unassisted death (p = 0.20) or survival during (p = 0.08) or after rehabilitation (short-term p = 0.38, long-term p = 0.40); however, not all levels of predictors were present in both classes at all survival stages, so we assessed classes separately for effects of study location, diel activity pattern, trophic level, and adult weight class (referred to as the full model). No factors were significant for either birds or mammals for survival to the end of rehabilitation (S3 Table). The log-odds of unassisted death during care for mammals was best explained by trophic level and adult weight class; omnivores had a significantly higher (p < 0.0001) death rate than carnivores (Table 3; Fig 2) (there was a significant association between trophic level and diel activity pattern (p = 0.025) and between trophic level and location (p = 0.046), so these combinations were excluded from models). Short-term post-release survival of mammals was best explained by two models including trophic level, location and diel activity with support based on AIC values, although no factors were significant (there was a significant association between adult weight class and diel activity (p = 0.0001), adult weight class and location (p < 0.001), and diel activity and location (p = 0.046), so these combinations were excluded). For long-term post-release survival of mammals, the full model had the best fit, although no predictors had a significant effect on survival (S3 Table).

**Table 3:** Summary of mixed-effects meta-regression models with the best fit and significant predictors of survival for bird and mammal classes for each survival stage.

**Unassisted death log-odds**						
**Aves (*I***^***2***^ **= 87.36)** ^**a**^	**Estimate**	**s.e.**	**Z-value**	**Lower**	**Upper**	**P-value**
Intercept ^b^	-1.76	0.13	-13.62	-2.02	-1.51	<0.001*
locationEurope	0.35	0.12	2.97	0.12	0.59	0.003*
locationNorthAmerica	0.75	0.13	5.96	0.50	1.00	<0.001*
locationOceania	-0.43	0.22	-1.92	-0.87	0.01	0.055
locationOther	0.14	0.26	0.55	-0.37	0.65	0.582
**Mammalia (*I***^***2***^ **= 97.56)**	**Estimate**	**s.e.**	**Z-value**	**Lower**	**Upper**	**P-value**
Intercept	-2.30	1.14	-2.02	-4.54	-0.07	0.043*
trophic_levelHerbivore	0.36	0.33	1.09	-0.28	1.00	0.274
trophic_levelOmnivore	1.12	0.33	3.36	0.47	1.78	0.001*
adult_weight_classMedium	0.79	1.12	0.71	-1.40	2.98	0.479
adult_weight_classSmall	0.31	1.11	0.28	-1.86	2.48	0.779
**Post-release short-term survival log-odds**						
**Aves (*I***^***2***^ **= 40.30)**	**Estimate**	**s.e.**	**Z-value**	**Lower**	**Upper**	**P-value**
Intercept	2.56	0.71	3.63	1.18	3.95	<0.001*
trophic_levelCarnivore	-1.95	0.59	-3.30	-3.10	-0.79	0.001*
trophic_levelHerbivore	0.80	0.94	0.85	-1.04	2.64	0.394
trophic_levelOmnivore	-0.31	0.74	-0.42	-1.76	1.13	0.671
diel_activityNocturnal	2.16	0.50	4.29	1.17	3.14	<0.001*
locationEurope	-2.16	0.52	-4.14	-3.18	-1.14	<0.001*
locationNorthAmerica	-2.01	0.51	-3.93	-3.01	-1.01	<0.001*
locationOceania	-0.62	0.80	-0.78	-2.18	0.94	0.438
**Post-release long-term survival log-odds**						
**Aves**	**Estimate**	**s.e.**	**Z-value**	**Lower**	**Upper**	**P-value**
**(I**^**2**^ **= 98.29) (AIC 198.69)** Intercept	1.78	2.09	0.85	-2.32	5.88	0.395
diel_activityDiurnal	-0.85	2.02	-0.42	-4.81	3.11	0.674
diel_activityNocturnal	-1.26	1.99	-0.63	-5.17	2.64	0.527
locationEurope	-1.60	0.87	-1.85	-3.30	0.09	0.064
locationNorthAmerica	-3.70	1.03	-3.59	-5.72	-1.68	<0.001*
locationOceania	-0.17	1.04	-0.16	-2.21	1.87	0.871
**(I**^**2**^ **= 97.77) (AIC 197.87)** Intercept	0.92	0.49	1.90	-0.03	1.88	0.058
trophic_levelHerbivore	0.93	0.86	1.07	-0.76	2.61	0.283
trophic_levelOmnivore	1.49	1.90	0.78	-2.23	5.21	0.433
locationEurope	-2.01	0.76	-2.62	-3.50	-0.51	0.009*
locationNorthAmerica	-3.59	0.93	-3.88	-5.41	-1.78	<0.001*
locationOceania	-0.80	0.87	-0.92	-2.52	0.91	0.357

^a^ *I*^*2*^ reports between-study heterogeneity

^b^ the mixed-effects meta-analysis function treats the first alphabetical factor level as a baseline with an estimate of zero; i.e., locationAfrica, trophic_levelApexPredator, diel_activityAnytime, adult_weight_classLarge

* P-values <0.05 indicate factor levels that are significantly different from zero

**Fig 3:**
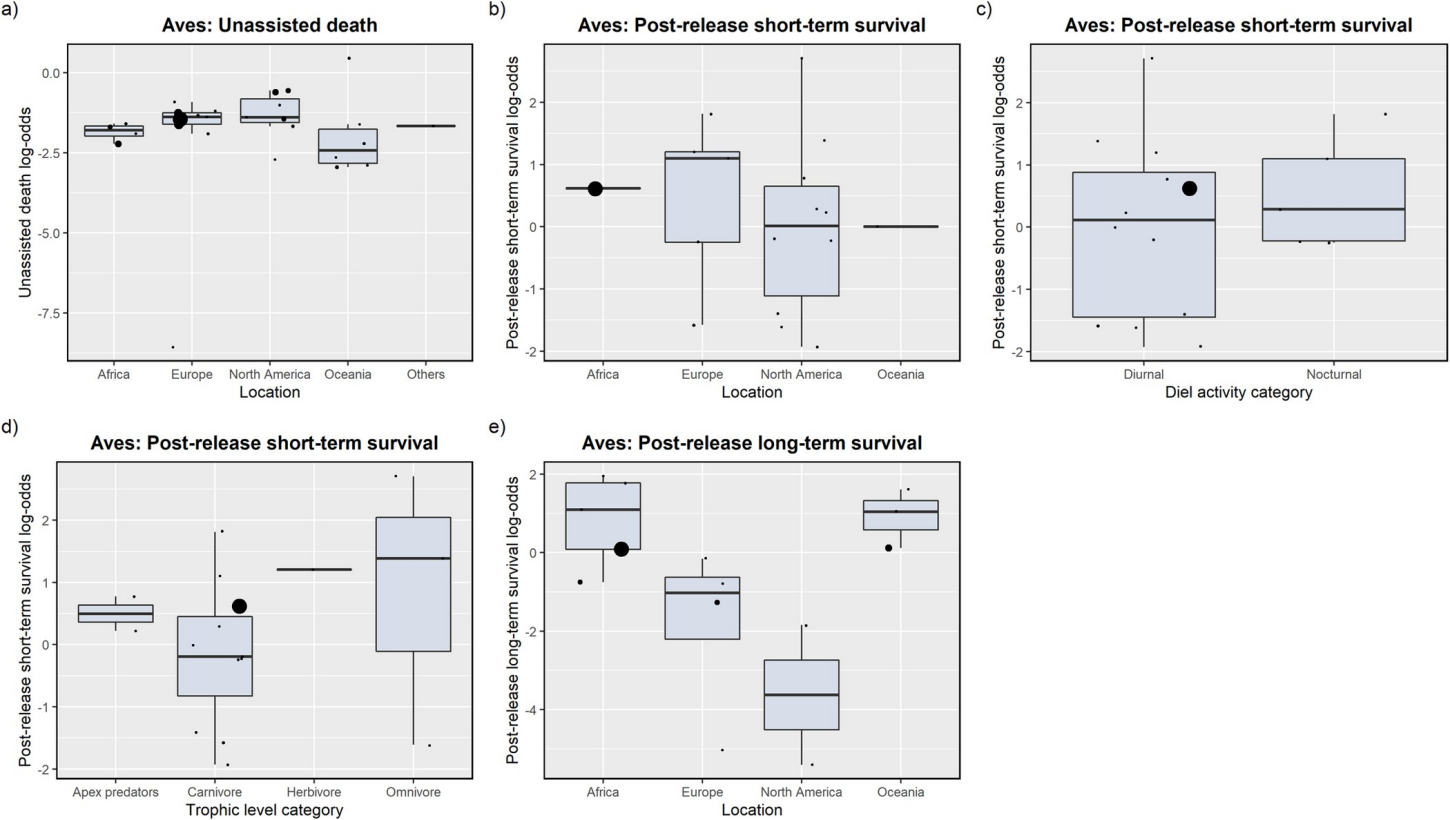
Distribution of log-odds across factors affecting unassisted death rates and short- and long-term post-release survival for avian species. Note that each point represents the log-odds of survival of one study-species combination, and the size of the point is proportional to the inverse variance of the log-odds (i.e. larger points have more weight).

Study location affected the unassisted death rate of birds; North America had the highest log-odds of death and Oceania had the lowest log-odds of death (Table 3). Mean probabilities of unassisted death were 10% Oceania, 15% Africa, 17% Others, 20% Europe, and 27% North America (Fig 3A). For short-term post-release survival, the model with trophic level, diel activity and location had the best fit for birds, although a large proportion of the variation came from sampling variation (*I*^*2*^ < 50%; Table 3). Studies in Africa had a higher survival probability than Europe, North America and Oceania (65%, 49%, 55% and 50%, respectively; Fig 3B), diurnal birds had lower survival probability than nocturnal birds (51% and 64%, respectively, Fig 3C), and carnivorous birds had lower survival probability than apex predators, herbivorous and omnivorous birds (45%, 63%, 77%, and 66%, respectively; Fig 3D). Two models including diel activity, location and trophic level had support based on AIC values for long-term survival of birds, and study location had a significant effect on survival (p < 0.0001; Table 3); North America had the lowest survival probability (6% North America compared with 31% Europe, 65% Oceania and 72% Africa; Fig 3E).

### Factors associated with survival in individual studies

In the reviewed articles, 18 included an experimental design to assess the outcomes of different methods of rehabilitation (e.g. hand-rearing diet, treatment regime, and soft versus hard release). Fifty-eight studies statistically assessed the effect of various factors on survival, and 38 included observations of factors that authors believed affected survival rates in their study (Table 4). Most factors were intrinsic to the individual or species (e.g. body size, age, and sex), or related to the interventions enacted by humans in the rescue, rehabilitation and release process (e.g. rescue protocol, habituation to humans, and release location) (Table 4).

**Table 4:** A summary of factors associated with wildlife survival during rehabilitation and after release, with the direction of effects (higher or lower probability of survival), and factors categorised into event-related, intrinsic or individual (inter-specific and intra-specific), intervention-related, release environment, and human-wildlife interface (see S5 Table for full list of species referenced).

Factor	Phase affected	Higher probability of survival after rescue	Lower probability of survival after rescue
**Event-related**			
** Reason for admission and associated diagnosis and severity**	Survival to release [32–58]	Less severe reason for rescue or diagnosis, e.g. uninjured orphans^a^	More severe reason for rescue or diagnosis, e.g. fracture^a^
	Survival post-release [34]	Less extensive oiling^a^	More extensive oiling^a^
** Size of disaster**	Survival to release [33, 59]	Major oil spill event i.e. greater search and recovery effort^a^[59]	Widespread events (e.g. heat stress) can overwhelm rehabilitator capacity^b^ [33]
** Season of admission**	Survival to release [54]	N/A^c^	Coincides with physiologically taxing events, such as breeding or moulting^a^
**Intrinsic or individual—inter-specific**			
** Body size**	Survival to release [60]	Larger body size^b^	N/A
** Migratory or not**	Survival post-release [60]	Non-migratory (i.e. no strain of migration soon after release)^b^	Migratory (i.e. become oiled far from breeding localities)^b^
** Behaviour**	Survival to release [61]	Resilient and easily adapts to captivity^b^	N/A
	Survival post-release [62]	Easily adapts to post-release environment^b^	N/A
**Intrinsic or individual—intra-specific**			
** Age**	Survival to release [6, 33, 36, 37, 44, 50, 53, 57, 59, 63–66]	Juveniles may present with less severe injuries such as orphaning, so have greater survival than adults^a^ [6, 33, 36, 37, 44, 50, 53, 57, 66]	Juveniles can have lower survival than adults^a^, often due to characteristics of their age such as moult, presence of an umbilicus (susceptible to infection) or differing fitness requirements [59, 63–65]
	Survival post-release [67, 68]	Juvenile pelicans had better survival than adults^a^ [68]	Juvenile (hand-reared) possums did not survive as long as adults^a^ [67]
** Sex**	Survival to release [47, 51, 69]	Male polecats were more likely to be released than females^a^ [69]	Male sparrowhawks were less likely to be released than females^a^ [51], male raptors were more susceptible to unassisted mortality than females [47]
	Survival post-release [34]	Male little penguins had higher survival rates than females^a^ [34]	N/A
** Bodyweight**	Survival to release [37, 54, 57, 59, 64, 70–72]	Heavier bodyweight at time of rescue^a^	Lower rescue mass and poor rescue condition^a^
	Survival post-release [34]	As above^a^	As above^a^
** Individual personality**	Survival post-release [73]	More exploratory individuals^a^	Less exploratory individuals^a^
** Brood size**	Survival to release [72]	Improved juvenile survival when larger brood is rescued^a^	N/A
** Physiological parameters**	Survival to release [41, 54]	Higher body temperature, higher packed cell volume and higher total plasma protein^a^ [41]	Low total plasma protein, low haematocrit^a^ [54]
** Activity pattern**	Survival to release [44]	Young diurnal raptors were admitted more frequently and had greater release rates than adults^a^	Adult nocturnal raptors were admitted more frequently than young birds^a^. Diurnal birds were more often treated for fractures than nocturnal birds^a^
**Intervention-related**			
** Personnel and facilities for rescue, transport, care, and release**	Survival to release [35, 36, 59, 60, 74–78]	Readily available and adequately equipped care facilities, trained personnel, and refined protocols^b^	Birds delivered by the public to the wildlife care centre (versus an animal collection officer or veterinarian)^b^^b^; time delay between event (e.g. oil spill) and rescue or veterinary treatment^b^
	Survival post-release [75, 79]	As above^b^	N/A
** Wildlife care centre**	Survival to release [36, 80]	Some centres had higher survival^a^, possibly due to increased levels of experience and appropriate triage and treatment regimes	Some centres had lower survival^a^, possibly due to lower levels of experience and ineffective or inappropriate triage and treatment regimes
** Choice of hand-raising diet**	Survival to release [71, 81]	Artificial milk-replacer had greater survival than fish-formula^a^, and a commercial milk-replacer was better than another brand^a^	N/A
** Maintenance of bodyweight**	Survival post-release [74, 82–85]	Sufficient fat reserves or heavier mass on release^a^^b^	Weight loss in migratory birds^b^
** Treatment regimens**	Survival to release [6, 35, 66, 86]	Individuals with a better response to treatment^a^; disease management and ancillary treatment^a^	Treatment based on clinical signs rather than diagnostic tests^b^; incorrect drug dosages given by rehabilitators^b^, inappropriate treatments^b^
	Survival post-release [66, 68, 87–89]	Appropriate disease management^b^	Inadequate oil removal procedures^b^; failure to eliminate pathogen before release^b^
** Habituation to humans**	Survival to release [6]	Less human-imprinted animals are more likely to survive^a^ [6]	N/A
	Survival post-release [6, 34, 67, 73, 85, 90–93]	Shorter periods of rehabilitation may be better^b^ [67], although duration did not affect little penguin survival^a^ [34]	Loss of wild behaviours such as predator avoidance and disruption of social development due to human habituation^a^^b^, although habituation was not related to survival in deer^a^ [90]
** Hunting and wild behaviour training**	Survival to release [78]	Construction of a pre-release flight tunnel for raptors^b^ [78]	N/A
	Survival post-release [91, 94–97]	Provision of suitable hunting training^b^	Lack of pre-release training to navigate situations likely to be encountered in the wild^b^; the mother-fawn relationship is essential, so hand-reared fawns lacked traits required for survival^b^ [97]
** Formation of social groups in captivity**	Survival post-release [93, 98, 99]	Formation of social groups and surrogate mentor females provided for juveniles^a^^b^.	Lack of group cohesiveness prior to release^b^.
** Readiness for release**	Survival post-release [40, 68, 79, 84, 85, 89, 100, 101]	Yearling age improves survival in bear cubs^a^; adequate waterproofing and bouyancy for water birds^b^	Released with unresolved ailments or long-term effects from reason for rescue^b^
**Release environment**			
** Timing of release**	Survival post-release [67, 76, 79, 82, 100]	Release during the non-breeding season^a^; mild weather^b^; high prey or food availability^b^	Majority of hedgehog mortalities occurred during spring when they are most active^a^ [82]
** Release method**	Survival post-release [60, 62, 82, 97, 98, 101–104]	Release to a wild flock or known congregation area^b^; release of female macropods in groups with other female rearing companions^b^; soft release^b^ [97, 101, 104]^a^; release close to breeding locations^b^	Hard released Asiatic black bears had shorter survival than soft-released bears^a^ [104]
** Familiarity of habitat**	Survival post-release [13, 34, 67, 90, 105–107]	Familiar release habitat may not be necessary for all species [34, 67, 105]^b^	Unfamiliar release habitat^b^
** Habitat quality**	Survival post-release [67, 92, 95, 107–113]	Unfamiliar habitat may be suitable while there are sufficient food trees and the carrying capacity has not been exceeded^b^ [67, 108]	At or near carrying capacity^b^; reduced canopy continuity following bushfires^b^; competition and attacks from conspecifics^b^; the need to immediately travel long distances^b^; the presence of illegal hunting activity and proximity to dwellings and roads^b^.
** Predators**	Survival post-release [8, 22, 95]	Control or absence of predators^b^	Presence of predators^b^
**Human-wildlife interface**			
** Increasing human population and habitat fragmentation**	Survival post-release [65, 114]	N/A	Stress associated with bushfires and habitat fragmentation may be contributing to disease in koalas^b^
** Hunting activities**	Survival post-release [84]	N/A	Survival rates of bears reflect their permitted hunting pressure^b^
** Urban expansion**	Survival post-release [84]	N/A	Increasing encounters in recolonised areas results in more illegal kills^b^

^a, b^ Factors identified by statistical or other inference (denoted by superscript a and b, respectively) that affected survival to release or survival post-release.

^c^ N/A indicates fields where no relevant data were presented. If survival to release or survival after release do not appear under phase affected for a given factor this indicates that no studies assessed that phase.

### Post-release survival of rehabilitated and control animals

Only 18 studies incorporated a control group, which was usually a wild cohort that did not require rehabilitation. Most studies showed either reduced survival for rehabilitated animals compared with control groups, or similar outcomes (Table 5). Oiling in particular appears to cause a large decrease in survival after rehabilitation, highlighted in the study by De La Cruz et al. [40] where oiled rehabilitated surf scoters (*Melanitta perspicillata*) showed survival of only 14.3% compared with 49.8% for unoiled non-rehabilitated scoters at five months, while unoiled scoters rehabilitated for other reasons had 77.2% survival.

**Table 5:** Post-release survival of rehabilitated and control groups of various wildlife species grouped by survival pattern between the two groups.

Species	Rehabilitated group survival	Control group survival
**Rehabilitated group survival less than control group**		
Common ringtail possums (*Pseudocheirus peregrinus*) [106]	101 days	182 days
Little penguins (*Eudyptula minor)* [34]	59% (Ninth Island), 44% (Low Head)	77% (Ninth Island), 50% (Low Head)
Brown pelicans (*Pelecanus occidentalis californicus*) [68]	9% at 2 years	53% at 2 years
Common murres (*Uria aalge)* [79]	45% at 60 days	92% at 60 days
Common murres [12]	39 days	485 days
Cape vultures (*Gyps coprotheres*) [7]	74.8% annual survival	91.3% annual survival
American coots (*Fulica americana*) [115]	49% at 4 months	76% at 4 months
Surf scoters (*Melanitta perspicillata*) [40]	14.3% at 5 months	49.8% at 5 months
**Rehabilitated group survival greater than control group**		
Hedgehogs (*Erinaceus europaeus*) [83]	73.1% at 8 weeks	63.6% at 8 weeks
Surf scoters [40]	77.2% at 5 months	49.8% at 5 months
**Rehabilitated group survival similar to control group**		
Sea otters (*Enhydra lutris*) [93]	71% at 1 year	75% at 1 year
Koalas (*Phascolarctos cinereus*) [22]	58% annual survival	67% annual survival
Carnaby’s cockatoos (*Zanda latirostris*) [98]	73% annual survival	61% - 69% annual survival
Hedgehogs [82, 116]	57% at 38 days	50% at 38 days
Shorebirds [74]	50% at 6 months	52% at 6 months
Western gulls (*Larus occidentalis*) [76]	100% at 6 months	90% at 6 months
Cape gannets (*Morus capensis*) [60]	86% (Malgas Island), 88% (Bird Island) annual survival	88% (Malgas Island), 90% (Bird Island) annual survival
Peregrine falcons (*Falco peregrinus)* [117]	14% at 1 year	10–11% at 1 year

## Discussion

This systematic review supports our hypothesis that wildlife survival during rehabilitation and post-release is species- and context-specific. Most studies in this review were from Australia, Europe, North America and southern Africa. Meta-analysis demonstrated effects of species’ diel activity type, trophic level, and location of the study on survival, but not adult weight, supporting some but not all our hypotheses. Study location was a strong predictor of death in care and survival short- and long-term post-release for birds. There are various potential explanations for the effect of location, including differing triage protocols and therefore frequency of euthanasia, impacts of different threats in the environment, and varying perceptions towards the value of wildlife around the world [3, 118, 119]. The reason for rescue and associated severity of diagnosis were strong predictors of survival to release, and in some cases, post-release survival. Our results synthesised five clear categories of factor that can impact survival outcomes for rescued wildlife and that must be addressed in rescue, rehabilitation and release protocols. These factors relate to the event, individual animal, intervention, release environment, and the human-wildlife interface. Oil spill events appear to have stimulated global wildlife rehabilitation research efforts, with 15 articles published from five countries, and generally result in low rates of survival. Only two articles (both in Australia) assessed the survival of rehabilitated wildfire-affected animals, and showed that they had low to moderate rates of survival [22, 109]. Overall, the number of studies that included an experimental approach or control to assess factors affecting survival was low.

### Factors affecting survival during and after rehabilitation

Unassisted deaths in care can act as an indicator of ineffective triage criteria and appropriate treatment and husbandry protocols, as these are animals that die without euthanasia. The rates of unassisted death varied depending on the study location for birds, and by trophic level for mammals. This variation could indicate that the decision to euthanise is made sooner in Oceania compared with North America, or that threats in North America are more likely to result in unpredictable death in care. The only omnivorous mammals with unassisted death rates recorded in the meta-analysis were raccoon dogs (*Nyctereutes procyonoides*) and European hedgehogs (*Erinaceus europaeus*), both with relatively high death rates. Hedgehogs that were admitted due to trauma, parasite infections and vehicle collisions had very low recovery rates, and road casualties died very quickly in care [56, 61]. Raccoon dogs treated in Japan for severe *Sarcoptes scabiei* infections experienced 57.4% and 38.9% unassisted death rates for two groups given different treatment regimens [86], allowing the researchers to determine the best treatment to reduce future unassisted deaths. The greatest death rate recorded in the review was 61.3% for little penguins (*Eudyptula minor)* rescued after an oil spill event in Australia [35]. Most mortalities occurred within the first 12 days, and were attributed to the degree of oiling, the amount of oil ingested, the low body weight of penguins on arrival, and inappropriate cleaning techniques used by inexperienced and unsupervised volunteers [35]. An oil spill five years later showed greatly improved survival rates for little penguins, with only 5% unassisted deaths [34], highlighting the importance of evaluating outcomes and refining protocols over time [77].

In the reviewed articles, there were several large mammalian species with high short-term post-release survival rates. In our meta-analysis, large species included Asiatic black bears (*Ursus thibetanus*), American black bears (*U*. *americanus*) and brown bears (*U*. *arctos*) in North America and white rhinoceros (*Ceratotherium simum*) in South Africa. Their high rate of survival could be associated with the success of the captive rearing process, as most of these animals entered rehabilitation as orphans [84, 108, 120], or a reduced risk of predation conferred by their size. In the long-term post-release, birds in North America had particularly low rates of survival, relating to studies of the peregrine falcon (*Falco peregrinus*) and brown pelican (*Pelecanus occidentalis californicus*). Anderson et al. [68] determined that rescue and treatment after oiling did not restore pelicans to normal survivability; however, the 14% survival rate of peregrine falcons was similar to non-rehabilitated peregrines in the same population [117]. As stated by Morris et al. [13], “rehabilitation cannot confer immortality” (pg. 65), and released rehabilitated animals are susceptible to the same threats as their wild counterparts [91], yet not always equally. For example, rehabilitated and wild ringtail possums (*Pseudocheirus peregrinus*) in Australia faced the same predation pressures, however, translocated rehabilitated possums were at a disadvantage in unfamiliar territory and initially had lower survival rates [106].

Few studies have experimentally assessed factors influencing wildlife survival during rehabilitation and release [1], yet many of the reviewed articles retrospectively assessed or made observations of factors that influenced survival in their study. The reason for admission and the associated severity of diagnosis were both predictors of survival to release in many studies across a broad range of species, particularly birds [42, 44, 58]. However, this was not always the case. For example, the initial cause for rescue had no effect on wombat (*Vombatus ursinus*) survival during rehabilitation, where age and response to treatment were predictive of survival instead [6]. In many avian studies, especially on raptors, the main reasons for admission to care were trauma and orphaned young, with trauma resulting in lower release rates, while raising orphaned young was relatively successful [48]. The large proportion of carnivorous/pescatarian birds rescued due to oiling may have contributed to the low short-term post-release survival of birds revealed by our meta-analysis.

Our review found that intrinsic traits of species or individuals can affect survival outcomes, yet traits of importance vary with the species and type of injury they sustain. For example, the large body size and non-migratory nature of Cape gannets (*Morus capensis*) may have contributed to higher release rates after oiling than for smaller oiled bird species [60]. Age and bodyweight upon entry to care were often correlated with survival to release, and in some studies, survival differed by sex. Larger body sizes may contribute to higher release rates in some cases by conferring a degree of robustness to the animal, or through increased effort contributed to rehabilitating larger species considered to be charismatic megafauna [121]. Although some physiological parameters were associated with survival, it can be impossible to define a cut-off measure to guide triage protocols [70].

The reviewed studies reported many intervention-related aspects of rescue, transport, treatment, and release methods that affect survival pre- and post-release. Habituation to humans and the associated loss of wild behaviours such as predator avoidance can result in poor survival [6, 73, 85, 90–92]. Therefore, shorter periods of rehabilitation may be better [67], yet in this time it is critical to teach hunting, foraging and wild behaviours to support survival [91, 94, 95]. Depending on species’ social behaviour, it may be important to form and release social groups together [112]. The provision of mentor animals could also provide benefits for animals such as deer, as the mother-fawn relationship has been shown to be essential for survival [97]. A veterinary examination prior to release is important in assessing readiness in terms of appropriate age, physical fitness, independence and recovery from disease or injury, and requirements will vary between species for optimising survival [68, 89, 101].

Several factors relating to the release environment were found to influence survival of rehabilitated wildlife, including the timing of release, release method, quality of the release habitat, and presence of predators. Responses varied among species. For example, soft release improved survival of Asiatic black bears [104], but not kangaroos and tawny owls [102, 122]. Habitat familiarity and quality also can be critical for some species’ long-term survival [108]. Thus, an unfamiliar environment contributes to low survival rates for possums, hedgehogs and deer [13, 90, 106, 107]. For koalas, habitat quality is more important than habitat familiarity [67], possibly due to their specialist feeding habits. A few studies identified effects of the human-wildlife interface on survival [44]. For example, survival rates of bears reflected hunting pressures, with increased numbers of encounters in recolonised areas resulting in more illegal kills by local residents [84].

### Limitations of the papers in this review

It is possible that relevant articles were missed in our search, particularly if they were published in another language, which may be why some regions were not represented in our results. Our analysis also indicated some publication bias. However, asymmetry in funnel plots does not always reflect publication bias and can result from other factors such as poor methods leading to exaggerated effects in smaller studies [31]. Few studies in this review included a control group, even though comparing survival with a control group is beneficial to assess whether rehabilitated wildlife is disadvantaged post-release. Lunney et al. [22] found that if they had examined only rehabilitated burnt koalas, their project would have been determined a failure due to the low survival rates, yet survival was similar to that of unburnt koalas in the same area. Another confounding factor was the different post-release monitoring methods, durations and measures of survival presented by reviewed articles. Some authors presented minimum percentages of animals known to be alive, while others calculated an annual survival rate or mean days survived, and there were often large numbers of individuals unaccounted for due to emigration from the monitoring area, failure of tracking collars, or early conclusion of fieldwork [102]. Several retrospective studies utilising wildlife rescue centre and rehabilitation databases acknowledged the poor quality and inconsistency of the data recorded [2, 3, 32, 33]. We note that some zoos contribute to rehabilitation research through their wildlife hospitals and other partnerships. However, these outcomes were not specifically searched for via zoo webpages, as relevant articles could have been detected in Google Scholar and conference proceedings searches (outlined in S1 Table).

### Lessons from reintroduction biology

There is a wealth of published studies on conservation translocation and reintroduction programs with varying levels of success [5, 14], which could be used to improve release protocols after rehabilitation. Some wildlife rehabilitation standards and guidelines include a requirement that rescued wildlife be returned to the location where they were found, if possible. Yet, with areas of suitable habitat diminishing [123, 124], or when the reason for rehabilitation is habitat loss (for example, catastrophic bush fires), policies for the translocation of rescued wildlife may need to be considered where survival will not be negatively affected.

Batson et al. [125] synthesised 30 techniques that have been used in translocation programs to influence post-release survival, separated into Animal Focused Tactics and Environmental Focused Tactics. The factors associated with survival presented in Table 4 align with many of these tactics, and as such could be used as a checklist prior to release of rescued wildlife and as a guide for future research priorities in rehabilitation programs. Research should be conducted to support best practice recommendations for each of these tactics, and we recommend that wildlife rescue organisations ensure that they educate their rehabilitators on these tactics (where data are available) for species in their region.

Environmental preconditioning in the form of predator control is an important consideration, particularly for translocation programs, given the large number of failures attributed to predation [14]. Our results show that introduced and native predators also played a role in mortalities of released rehabilitated wildlife. There is evidence to support the benefits of protection from predators (via wildlife training or use of a fence), predator control efforts (e.g. baiting or shooting), and absence of predators [126–129]. It would be beneficial for environmental managers to engage with wildlife rehabilitation organisations and provide data about introduced predator and conspecific densities in surrounding habitat, and any intended control programs, so that suitable release locations can be appropriately identified.

Conservation translocations follow guidelines set by the International Union for Conservation of Nature [130], which state that post-release monitoring is an essential part of a responsible conservation translocation with data collected on survival, reproduction and dispersal. However, post-release monitoring by wildlife rehabilitators is often limited due to lack of funds, lack of expertise, and onerous requirements for state permission [19]. This is where collaboration between university and government researchers and wildlife rehabilitation organisations can provide great benefits. GPS tracking technology would assist post-release monitoring, and rapid advancements in technology now allow access to devices of smaller size and greater battery duration at low cost [5].

### Recommendations for wildlife rehabilitation and future directions

Adequate resources for rapid rescue responses are key to improving survival rates of wildlife, particularly after severe or widespread incidents [33, 59]. After the Black Summer bushfires in Australia there were cases where wildlife rescuers could not access fire grounds due to safety concerns or lack of support [15], highlighting the need for appropriate emergency response plans and resources [23]. It is also likely that veterinarians will encounter a larger volume and diversity of wildlife than they are accustomed to during disaster events. As such, it is vital to develop advice and support services for veterinarians.

Whether or not wildlife rehabilitation contributes to conservation outcomes is debated and lacks evidence [1], yet it will continue to be practiced around the world and likely play a role in the persistence of local wildlife populations following increasingly frequent and severe environmental disasters [20, 21]. The need to incorporate wildlife rescue into broader disaster response plans is gaining traction, with some organisations facilitating improvements to disaster preparedness [131] and developing wildlife first aid guidelines [132]. Rescued wildlife is exposed to the stress of the initial adverse event, in addition to stress occasioned by transport, treatments, captivity and release [114]. The potential distress experienced by animals needs to be pragmatically weighed with the benefits of survival for the individual, population and species. If animals are released with a reduced likelihood of survival this presents a potentially serious welfare concern if they are unable to adapt or are more susceptible to threats than wild counterparts. It is vital that research continues to develop our understanding of basic biology and husbandry requirements of native wildlife [133], along with factors associated with survival at all stages of rehabilitation. Our comparison of post-release survival rates between study and control groups has highlighted the value of including a comparison with a wild cohort in future studies. The factors highlighted by this review and summarised in Table 4 should be used as a framework to guide the development and revision of species-specific and evidence-based rescue and treatment protocols globally. With these robust protocols, veterinarians and rescue organisations can continue to minimise animal suffering and maximise the effectiveness of rehabilitation programs in an environment affected by climate change and urban expansion. Threat mitigation must also be prioritised to reduce the need for wildlife rescue in the first place.

## Supporting information

S1 Checklist (DOCX)

S1 Table Summary of the systematic search methods and numbers of articles returned. (DOCX)

S2 Table Publication bias funnel plot analysis, eggers test output and trim-and-fill analysis output. (DOCX)

S3 Table Summary of mixed-effects meta-regression models with the best fit but no significant predictors of survival. (DOCX)

S4 Table A summary of reviewed articles and the survival measures reported for mammals and birds during care, and in the short- and long- term post release. (DOCX)

S5 Table A summary of factors associated with wildlife survival during rehabilitation and after release, including relevant species and references. (DOCX)

S6 Table Systematic review article data used for meta-analysis. (XLSX)
