# Supplementary material for: A systematic review of factors affecting wildlife survival during rehabilitation and release
Source: PLoS One. 2022 Mar 17;17(3):e0265514. doi: 10.1371/journal.pone.0265514 (PMC8929655; doi:10.1371/journal.pone.0265514)
Supplement: S1 Checklist — (DOCX) [file pone.0265514.s001.docx]

| **Section and Topic** | **Item #** | **Checklist item** | **Location where item is reported** |
| --- | --- | --- | --- |
| **TITLE** | | |  |
| Title | 1 | Identify the report as a systematic review.  A systematic review of factors affecting wildlife survival during rehabilitation and release | Line 1-2 |
| **ABSTRACT** | | |  |
| Abstract | 2 | See the PRISMA 2020 for Abstracts checklist. | L12-31 |
| **INTRODUCTION** | | |  |
| Rationale | 3 | Describe the rationale for the review in the context of existing knowledge.  “For these reasons, wildlife rehabilitation is likely to continue, and assessments of the factors affecting wildlife survival during rehabilitation and release can help to inform future directions.”  “As such, it is valuable to assess current survival rates and factors associated with the success of rehabilitation of rescued wildlife.”  “We focused on mammals and birds as these classes are commonly rescued and rehabilitated, with survival data subsequently reported in the literature.” | L44-46, 71-72, 74-76 |
| Objectives | 4 | Provide an explicit statement of the objective(s) or question(s) the review addresses.  “We used a systematic approach to review survival rates of native mammals and birds during rehabilitation and post-release to determine factors associated with survival.”  “The effects of a range of intrinsic and extrinsic factors on survival were evaluated to develop a framework of key considerations for wildlife rehabilitation, and to guide future research on best-practice rehabilitation methods.” | L73-74, 76-78 |
| **METHODS** | | |  |
| Eligibility criteria | 5 | Specify the inclusion and exclusion criteria for the review and how studies were grouped for the syntheses.  “Full texts were then reviewed against selection criteria as follows: research was conducted on native mammals or birds that entered care for any reason; the sample size and a survival measure and timeframe (e.g. number of mortalities, annual survival rate, minimum known alive) during rehabilitation or post-release were reported; and the article (or abstract) was available online and in English.”  “One reviewer created a summary of study characteristics for each article detailing study species, sample size, study location, reason for entry into care, percentage of unassisted deaths in care (i.e. excluding euthanasia), percentage survival to release from care, short- and long-term survival post-release, factors reported as affecting survival, and causes of mortality. “  “We used the Encyclopedia of Life (https://eol.org/) to categorise species according to class (Aves, Mammalia), diel activity pattern (any time, crepuscular, diurnal, nocturnal), average adult weight (small < 5.5 kg, medium 5.5 – 100 kg, large > 100 kg) and trophic level (herbivores [primary consumers], omnivores and carnivores [secondary and tertiary consumers], apex carnivores).” | L92-96, 100-103, 108-111 |
| Information sources | 6 | Specify all databases, registers, websites, organisations, reference lists and other sources searched or consulted to identify studies. Specify the date when each source was last searched or consulted.  “Online databases Scopus and Web of Science were searched along with relevant conference proceedings, reference lists of selected articles (backwards search) and Google Scholar. The literature search was completed by H. Cope over a 3-month period from January to March 2021, and included 50 journals” | L82-85 |
| Search strategy | 7 | Present the full search strategies for all databases, registers and websites, including any filters and limits used. | Appendix S1 |
| Selection process | 8 | Specify the methods used to decide whether a study met the inclusion criteria of the review, including how many reviewers screened each record and each report retrieved, whether they worked independently, and if applicable, details of automation tools used in the process.  “As thousands of articles were returned, they were sorted by relevance then searched until there were 50 consecutive non-selected articles. The online systematic review tool, SysRev, (sysrev.com) was used to reduce the returned articles based on title, abstract and keywords. Full texts were then reviewed against selection criteria by one reviewer as follows:” | L89-92 |
| Data collection process | 9 | Specify the methods used to collect data from reports, including how many reviewers collected data from each report, whether they worked independently, any processes for obtaining or confirming data from study investigators, and if applicable, details of automation tools used in the process.  “One reviewer created a summary of study characteristics for each article…”  “We used the Encyclopedia of Life (https://eol.org/) to categorise species according to class (Aves, Mammalia), diel activity pattern (any time, crepuscular, diurnal, nocturnal), average adult weight (small < 5.5 kg, medium 5.5 – 100 kg, large > 100 kg) and trophic level (herbivores [primary consumers], omnivores and carnivores [secondary and tertiary consumers], apex carnivores).” | L100, 108-111 |
| Data items | 10a | List and define all outcomes for which data were sought. Specify whether all results that were compatible with each outcome domain in each study were sought (e.g. for all measures, time points, analyses), and if not, the methods used to decide which results to collect.  “One reviewer created a summary of study characteristics for each article detailing … percentage of unassisted deaths in care (i.e. excluding euthanasia), percentage survival to release from care, short- and long-term survival post-release, factors reported as affecting survival, and causes of mortality. Post-release survival was categorized as short-term (< 6 months) or long-term (> 6 months) to minimise bias between study outcomes.”  “The reasons for entry into care and causes of post-release mortality were grouped into 3 categories - anthropogenic, environmental, and non-specific (those that could not be attributed) - and reported as frequencies. Factors affecting survival during rehabilitation or after release were categorised as being related to the event that precipitated entry into care (e.g. severity and type of injury), intrinsic or individual traits (e.g. body size, behaviour and age), intervention (e.g. rescue protocols, choice of diet and pre-release training), release environment (e.g. timing of release, release method and habitat quality), and human-wildlife interface (e.g. hunting activity and urban expansion), and summarised.” | L100-105, 120-127 |
|  | 10b | List and define all other variables for which data were sought (e.g. participant and intervention characteristics, funding sources). Describe any assumptions made about any missing or unclear information.  “One reviewer created a summary of study characteristics for each article detailing study species, sample size, study location, reason for entry into care, …. | L100-101 |
| Study risk of bias assessment | 11 | Specify the methods used to assess risk of bias in the included studies, including details of the tool(s) used, how many reviewers assessed each study and whether they worked independently, and if applicable, details of automation tools used in the process.  “Publication bias can exist where small studies with small effect sizes are not published or there is selective reporting within studies. We tested for bias in METAFOR by creating a funnel plot of effect size versus sampling variance of the effect size for each survival measure (Sterne & Egger 2001). Egger’s test was used for funnel plot asymmetry and trim-and-fill analysis (Harrer et al. 2021) was used to estimate magnitude of publication bias.” Completed by one reviewer | L148-152 |
| Effect measures | 12 | Specify for each outcome the effect measure(s) (e.g. risk ratio, mean difference) used in the synthesis or presentation of results.  “A mixed-effects meta-regression model in the METAFOR package was used to assess the relationship between survival and characteristics of the study species. Species was included as a random effect to account for multiple studies on the same species. Effect sizes were weighted by the sample size due to a lack of reported error measures for most articles (survival was generally reported as percentage known alive), based on the expectation that variance will decrease with larger sample sizes. Survival rates and sample sizes were then used to calculate log-odds of survival. Survival was initially compared between Aves and Mammalia for each stage of rehabilitation, being the unassisted death rate in care (i.e. deaths not resulting from euthanasia), survival to the end of rehabilitation (i.e. release to the wild or long-term captivity), short-term survival post-release, and long-term survival post-release. Each class was then assessed separately to determine the effect of factors hypothesised to effect survival including study location (Oceania [and Asia], North America, Europe [and UK], Africa and Outliers [Middle East, Southern America]) and species' diel activity pattern, adult weight class and trophic level at each stage of rehabilitation. All combinations of predictors were modelled and Akaike’s Information Criterion (AIC) (Harrer et al. 2021) was used to select the best model with the lowest AIC value by ≥ 2 points. Where no model satisfied this criterion, the most parsimonious model (least number of predictors) within 2 points of the lowest AIC value was selected. Between-study heterogeneity was reported as *I^2^* (Viechtbauer 2021)*.* Probabilities of survival were calculated as a back-transformation of log-odds for single predictor models for simplicity of interpretation.” | L129-147 |
| Synthesis methods | 13a | Describe the processes used to decide which studies were eligible for each synthesis (e.g. tabulating the study intervention characteristics and comparing against the planned groups for each synthesis (item #5)).  “Survival was initially compared between Aves and Mammalia for each stage of rehabilitation, being the unassisted death rate in care (i.e. deaths not resulting from euthanasia), survival to the end of rehabilitation (i.e. release to the wild or long-term captivity), short-term survival post-release, and long-term survival post-release.” Studies were included based on the phase of survival that they assessed: “Each class was then assessed separately to determine the effect of factors hypothesised to effect survival including study location (Oceania [and Asia], North America, Europe [and UK], Africa and Outliers [Middle East, Southern America]) and species' diel activity pattern, adult weight class and trophic level at each stage of rehabilitation.” | L135-138, 138-142 |
|  | 13b | Describe any methods required to prepare the data for presentation or synthesis, such as handling of missing summary statistics, or data conversions.  “Effect sizes were weighted by the sample size due to a lack of reported error measures for most articles (survival was generally reported as percentage known alive), based on the expectation that variance will decrease with larger sample sizes.” | L132-134 |
|  | 13c | Describe any methods used to tabulate or visually display results of individual studies and syntheses.  “One reviewer created a summary of study characteristics for each article…” results were tabulated. The result format was not conducive to creation of visual representations i.e. no measures of error as survival was generally reported as a percentage known to be alive. | L100 |
|  | 13d | Describe any methods used to synthesize results and provide a rationale for the choice(s). If meta-analysis was performed, describe the model(s), method(s) to identify the presence and extent of statistical heterogeneity, and software package(s) used.  “Statistical analyses were performed in R (version 4.0.5) (R Core Team 2021). A mixed-effects meta-regression model in the METAFOR package was used to assess the relationship between survival and characteristics of the study species.”  “Between-study heterogeneity was reported as *I^2^* (Viechtbauer 2021)” | L129-131, 145 |
|  | 13e | Describe any methods used to explore possible causes of heterogeneity among study results (e.g. subgroup analysis, meta-regression).  The binomial nature of the survival measure (alive/dead) and lack of error measure (generally no s.e. reported) made this analysis problematic. | N/A |
|  | 13f | Describe any sensitivity analyses conducted to assess robustness of the synthesized results.  The binomial nature of the survival measure (alive/dead) and lack of error measure (generally no s.e. reported) made this analysis problematic. | N/A |
| Reporting bias assessment | 14 | Describe any methods used to assess risk of bias due to missing results in a synthesis (arising from reporting biases).  “These sub-groups were considered sufficient to reduce risk of bias from individual studies. There were insufficient samples to further stratify studies based on methods used.” | L116-118 |
| Certainty assessment | 15 | Describe any methods used to assess certainty (or confidence) in the body of evidence for an outcome.  The binomial nature of the survival measure (alive/dead) and lack of error measure (generally no s.e. reported) made this analysis problematic. | N/A |
| **RESULTS** | | |  |
| Study selection | 16a | Describe the results of the search and selection process, from the number of records identified in the search to the number of studies included in the review, ideally using a flow diagram.  “The literature search yielded 5617 publications, of which 187 were initially selected; after reviewing the full texts, 112 articles satisfied all inclusion criteria (Fig. 1).” | L154-155 |
|  | 16b | Cite studies that might appear to meet the inclusion criteria, but which were excluded, and explain why they were excluded. | Fig.1 |
| Study characteristics | 17 | Cite each included study and present its characteristics. | Table 4, 5, Appendix S3 |
| Risk of bias in studies | 18 | Present assessments of risk of bias for each included study. | N/A |
| Results of individual studies | 19 | For all outcomes, present, for each study: (a) summary statistics for each group (where appropriate) and (b) an effect estimate and its precision (e.g. confidence/credible interval), ideally using structured tables or plots.  “Table 1. Number of published articles from each country or region reporting survival of wildlife during rehabilitation, post-release or during both phases.”  “S3 Table. A summary of reviewed articles and the survival measures reported for mammals and birds during care, and in the short- and long- term post release.” | Table 1, Appendix S3 |
| Results of syntheses | 20a | For each synthesis, briefly summarise the characteristics and risk of bias among contributing studies.  “Funnel plot analysis showed an estimated lack of 13 studies with large effect sizes for unassisted death in care (p = 0.0006) resulting in possible underestimation in our results, 6 missing studies with small effect sizes for both rehabilitation survival (p = 0.0038) and post-release short-term survival (p = 0.0328) resulting in possible overestimation, and no publication bias in long-term post-release survival (p = 0.1397; Appendix S2).” | L161-166 |
|  | 20b | Present results of all statistical syntheses conducted. If meta-analysis was done, present for each the summary estimate and its precision (e.g. confidence/credible interval) and measures of statistical heterogeneity. If comparing groups, describe the direction of the effect.  “There were no differences between classes for unassisted death (p=0.20) or survival during (p=0.08) or after rehabilitation (short-term p=0.38, long-term p=0.40); however, not all levels of predictors were present in both classes at all survival stages, so we assessed classes separately for effects of study location, diel activity pattern, trophic level, and adult weight class (referred to as the full model). No factors were significant for either birds or mammals for survival to the end of rehabilitation (S4 Table). The log-odds of unassisted death during care for mammals was best explained by trophic level and location; omnivores had a significantly higher (p<0.0001) death rate than carnivores (Table 3; Fig 2a). However, the near-zero *I^2^* value indicates this is an artefact of sampling variance, rather than true variation in the population. Short-term post-release survival of mammals was best explained by the full model (Table 3). Species in the small and medium weight classes had decreased log-odds of survival compared with the large weight class (p=0.028 and p=0.012, respectively; Table 3). Mean survival probabilities were 80% large, 54% medium and 60% small (Fig 2b). For long-term post-release survival of mammals, the full model had the best fit, although no predictors had a significant effect on survival (S4 Table).”  “Table 3. Summary of mixed-effects meta-regression models with the best fit and significant predictors of survival for bird and mammal classes for each survival stage.” | L187-200, Table 3 |
|  | 20c | Present results of all investigations of possible causes of heterogeneity among study results. | N/A |
|  | 20d | Present results of all sensitivity analyses conducted to assess the robustness of the synthesized results. | N/A |
| Reporting biases | 21 | Present assessments of risk of bias due to missing results (arising from reporting biases) for each synthesis assessed. | N/A |
| Certainty of evidence | 22 | Present assessments of certainty (or confidence) in the body of evidence for each outcome assessed. | N/A |
| **DISCUSSION** | | |  |
| Discussion | 23a | Provide a general interpretation of the results in the context of other evidence.  Throughout the discussion, for example “Large species had the highest short-term post-release survival rates for mammals. In our meta-analysis, large species included Asiatic black bears (*Ursus thibetanus*), American black bears (*Ursus americanus*) and brown bears (*Ursus arctos*) in North America and white rhinoceros (*Ceratotherium simum*) in South Africa. Their high rate of survival could be associated with the success of the captive rearing process, as most of these animals entered rehabilitation as orphans (Beecham et al. 2015; Clark et al. 2002; Smith et al. 2016), or have a reduced risk of predation conferred by their size.” and  “Our review found that intrinsic traits of species or individuals can affect survival outcomes, yet traits of importance vary with the species and type of injury they sustain. For example, the large body size and non-migratory nature of Cape gannets (*Morus capensis*) may have contributed to higher release rates after oiling than for smaller oiled bird species (Altwegg et al. 2008). This effect of body size is supported by our finding that mammals greater than 100 kg had greater survival than smaller species in the short-term post-release.” | L293-298, 319-324 |
|  | 23b | Discuss any limitations of the evidence included in the review.  Under the subheading “*Limitations of the papers in this review”*  “Our analysis indicated some publication bias. However, asymmetry in funnel plots does not always reflect publication bias and can result from other factors such as poor methods leading to exaggerated effects in smaller studies (Sterne & Egger 2001). Few studies in this review included a control group, even though comparing survival with a control group is beneficial to assess whether rehabilitated wildlife is disadvantaged post-release.”…”Another confounding factor was the different post-release monitoring methods, durations and measures of survival presented by reviewed articles.” | L350-358 |
|  | 23c | Discuss any limitations of the review processes used.  Under the subheading “*Limitations of the papers in this review”*  “We note that some zoos contribute to rehabilitation research through their wildlife hospitals and other partnerships. However, these outcomes were not specifically searched for via zoo webpages, as relevant articles could have been detected in Google Scholar and conference proceedings searches” | L363-365 |
|  | 23d | Discuss implications of the results for practice, policy, and future research.  “Adequate resources for rapid rescue responses are key to improving survival rates of wildlife, particularly after severe or widespread incidents”  “As such, it is vital to develop advice and support services for veterinarians.”  “It is vital that research continues to develop our understanding of basic biology and husbandry requirements of native wildlife (Legge et al. 2018), along with factors associated with survival at all stages of rehabilitation. Species-specific and evidence-based rescue and treatment protocols need to be developed or revised so that veterinarians and rescue organisations can continue to minimise animal suffering and maximise the effectiveness of rehabilitation programs in an environment affected by climate change and urban expansion. Threat mitigation must also be prioritised to reduce the need for wildlife rescue in the first place. ” | L401-402, 406-407, 415-422 |
| **OTHER INFORMATION** | | |  |
| Registration and protocol | 24a | Provide registration information for the review, including register name and registration number, or state that the review was not registered.  This systematic review was not registered, and a review protocol was not prepared. | Completed during submisison |
|  | 24b | Indicate where the review protocol can be accessed, or state that a protocol was not prepared.  This systematic review was not registered, and a review protocol was not prepared. | Completed during submisison |
|  | 24c | Describe and explain any amendments to information provided at registration or in the protocol.  This systematic review was not registered, and a review protocol was not prepared. | Completed during submisison |
| Support | 25 | Describe sources of financial or non-financial support for the review, and the role of the funders or sponsors in the review.  H.R.C. was supported by a postdoctoral fellowship funded by the Morris Animal Foundation and the Sydney School of Veterinary Science. | Completed during submisison |
| Competing interests | 26 | Declare any competing interests of review authors.  The authors declare no conflicts of interest. | Completed during submisison |
| Availability of data, code and other materials | 27 | Report which of the following are publicly available and where they can be found: template data collection forms; data extracted from included studies; data used for all analyses; analytic code; any other materials used in the review.  All references for articles included in this review and their reported survival measures are provided in Appendix S3. Other data and analytic code have not been made publicly available. | Completed during submisison |

*From:*  Page MJ, McKenzie JE, Bossuyt PM, Boutron I, Hoffmann TC, Mulrow CD, et al. The PRISMA 2020 statement: an updated guideline for reporting systematic reviews. BMJ 2021;372:n71. doi: 10.1136/bmj.n71

For more information, visit: <http://www.prisma-statement.org/>
