# Supplementary material for: A systematic review of factors affecting wildlife survival during rehabilitation and release
Source: PLoS One. 2022 Mar 17;17(3):e0265514. doi: 10.1371/journal.pone.0265514 (PMC8929655; doi:10.1371/journal.pone.0265514)
Supplement: S1 Table — (DOCX) [file pone.0265514.s002.docx]

**S1 Table. Summary of the systematic search methods and numbers of articles returned.**

| **Focus** | **Search Term** | **Scopus + Web of Science** | **Backwards search** | **Other** | **Total** |
| --- | --- | --- | --- | --- | --- |
| International rehabilitation^a^ | TITLE-ABS-KEY [or TS for Web of Science] ((wildlife OR fauna* OR mammal* OR bird* OR avian OR cockatoo* OR psittacine* OR raptor* OR parrot*) AND (rehab* OR rescue* OR care OR carer* OR recovery) AND (surviv* OR release* OR fate) AND NOT (australia*)). Excluded irrelevant subject areas and sorted by relevance then searched until there were 50 consecutive non-selected articles (reached 660 articles in Scopus, 780 articles in WoS, and 210 in Google Scholar). | 5180 unique articles returned  70 initially selected  41 passed criteria | 65 initially selected  35 passed criteria | The International Wildlife Rehabilitation Council symposiums^b^  2 initially selected  1 passed criteria  Google Scholar search  wildlife rehabilitation survival  48,400 returned  10 passed criteria | 87 |
| Australian rehabilitation | ^c^TITLE-ABS-KEY [or TS for Web of Science] ((australia* OR australasia* OR nsw OR "new south wales" OR sydney OR qld OR queensland OR "northern territory" OR vic OR victoria* or tasmania) AND (wildlife OR fauna* OR mammal* OR marsupial* OR monotreme* OR koala* OR kangaroo* OR possum* OR wombat* OR echidna* OR wallab* OR glider* OR bandicoot* OR bettong* OR quoll* OR macropod* OR dasyurid* OR antechinus OR potoro* OR dunnart* OR bird* OR avian OR cockatoo* OR psittacine* OR raptor* OR parrot*) AND (rehab*** OR rescue* OR care OR carer* OR recovery) AND (surviv* OR reintroduc* OR release* OR translocat* OR fate)) | 437 unique articles returned  30 initially selected  18 passed criteria | 3 initially selected  1 passed criteria | Australian Wildlife Rehabilitation Conference Proceedings^d^  15 initially selected  6 passed criteria  *Thesis repositories*^e^  1 initially selected  0 passed criteria | 25 |
| International rehabilitation after fire | TITLE-ABS-KEY [or TS for Web of Science] ((bushfire* OR wildfire* OR fire* OR burn*) AND (wildlife OR fauna* OR mammal OR animal*) AND (rehab* OR rescue* OR care OR carer* OR caring OR recover* OR save) AND NOT (lumber OR tree OR vegetation OR Australia*))  and  California Department of Fish and Wildlife fire rehabilitation (Given the severe wildfires experienced in California in recent years, e.g. 2018 California Camp Fire), Wildlife fire rehabilitation USA, Wildlife fire rehabilitation canada | 550 and 325 returned  0 selected |  | Google and Google Scholar  0 initially selected | 0 |

^a^ Note that the search was initially conducted with a view to compare Australian studies with international studies, prior to the focus becoming global in nature.

^b^ https://theiwrc.org/symposium. The International Wildlife Rehabilitation Council publishes findings in the Journal of Wildlife Rehabilitation, of which several articles were included via the search of Scopus and Web of Science.

^c^ South Australia and Western Australia were not included as search terms, because the included term Australia would return these, and the acronyms of SA and WA were not included as they would return many non-specific articles.

^d^ https://www.awrc.org.au/past-conferences.html

^e^ Sydney University Library, Open Access Theses and Dissertations, and ANU Open Access Theses.
