# Supplementary material for: A systematic review of factors affecting wildlife survival during rehabilitation and release
Source: PLoS One. 2022 Mar 17;17(3):e0265514. doi: 10.1371/journal.pone.0265514 (PMC8929655; doi:10.1371/journal.pone.0265514)
Supplement: S2 Table — (DOCX) [file pone.0265514.s003.docx]

**S2 Table. Publication bias funnel plot analysis, Eggers test output and trim-and-fill analysis output for unassisted death, rehabilitation survival, short-term post-release survival and long-term post-release survival.**

Filled circles represent included articles; open circles represent missing articles.

| **Unassisted Death**  Regression Test for Funnel Plot Asymmetry  Model: mixed-effects meta-regression model  Predictor: sampling variance  Test for funnel plot asymmetry: z = -3.4286, p = 0.0006  Trim-and-fill analysis  Estimated number of missing studies on the right side: 13 (SE = 4.2512)  Model Results:  estimate se zval pval ci.lb ci.ub  -1.1701 0.1012 -11.5580 <.0001 -1.3686 -0.9717 ***  Original regression estimate: -1.4551 | 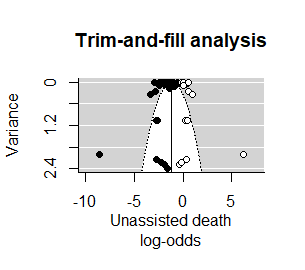 |
| --- | --- |
| **Rehabilitation survival**  Regression Test for Funnel Plot Asymmetry  Model: mixed-effects meta-regression model  Predictor: sampling variance  Test for funnel plot asymmetry: z = 2.8971, p = 0.0038  Trim-and-fill analysis  Estimated number of missing studies on the left side: 6 (SE = 5.1752)  Model Results with missing studies filled:  estimate se zval pval ci.lb ci.ub  0.1352 0.1120 1.2065 0.2276 -0.0844 0.3548  Original regression estimate: 0.2358 | 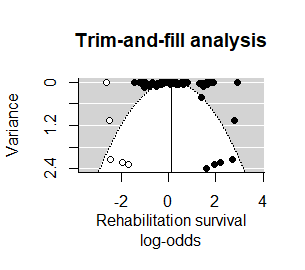 |
| **Post-release short-term survival**  Regression Test for Funnel Plot Asymmetry  Model: mixed-effects meta-regression model  Predictor: sampling variance  Test for funnel plot asymmetry: z = 2.1342, p = 0.0328  Trim-and-fill analysis  Estimated number of missing studies on the left side: 6 (SE = 4.3612)  Model Results:  estimate se zval pval ci.lb ci.ub  0.0580 0.1773 0.3271 0.7436 -0.2895 0.4055  Original regression estimate: 0.2984 | 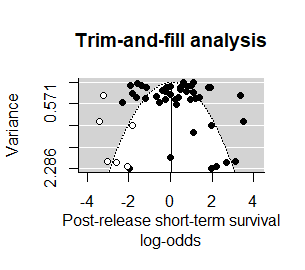 |
| **Post-release long-term survival**  Regression Test for Funnel Plot Asymmetry  Model: mixed-effects meta-regression model  Predictor: sampling variance  Test for funnel plot asymmetry: z = -1.4768, p = 0.1397 | 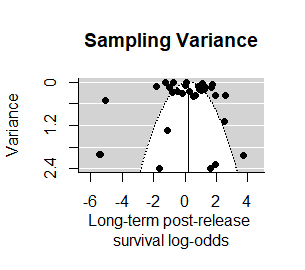 |
