# Supplementary material for: A systematic review of factors affecting wildlife survival during rehabilitation and release
Source: PLoS One. 2022 Mar 17;17(3):e0265514. doi: 10.1371/journal.pone.0265514 (PMC8929655; doi:10.1371/journal.pone.0265514)
Supplement: S3 Table — (DOCX) [file pone.0265514.s004.docx]

**S3 Table: Summary of mixed-effects meta-regression models with the best fit but no significant predictors of survival for bird or mammal classes for rehabilitation survival and post-release long-term survival**.

The mixed-effects meta-analysis function treats the first alphabetical factor level as a baseline with an estimate of zero i.e. locationAfrica, trophic_levelApexPredator, diel_activityAnytime, adult_weight_classLarge.

| **Rehabilitation survival log-odds** | | | | | | |
| --- | --- | --- | --- | --- | --- | --- |
| **Aves** | **Estimate** | **s.e.** | **Z-value** | **Lower** | **Upper** | **P-value** |
| intrcpt | -0.32 | 0.49 | -0.65 | -1.28 | 0.65 | 0.518 |
| trophic_levelCarnivore | 0.41 | 0.54 | 0.76 | -0.65 | 1.47 | 0.449 |
| trophic_levelHerbivore | -0.56 | 0.65 | -0.86 | -1.83 | 0.71 | 0.389 |
| trophic_levelOmnivore | 0.82 | 0.67 | 1.24 | -0.48 | 2.13 | 0.217 |
| locationEurope | 0.07 | 0.10 | 0.70 | -0.13 | 0.27 | 0.486 |
| locationNorthAmerica | 0.05 | 0.10 | 0.46 | -0.15 | 0.25 | 0.643 |
| locationOceania | 0.18 | 0.13 | 1.32 | -0.08 | 0.44 | 0.187 |
| locationOutlier | 1.68 | 0.25 | 6.64 | 1.18 | 2.17 | <.0001 |
| **Mammalia** | **Estimate** | **s.e.** | **Z-value** | **Lower** | **Upper** | **P-value** |
| intrcpt | 1.81 | 1.19 | 1.52 | -0.53 | 4.15 | 0.130 |
| diel_activityCrepuscular | -0.60 | 0.68 | -0.89 | -1.94 | 0.73 | 0.375 |
| diel_activityDiurnal | 0.52 | 0.75 | 0.70 | -0.94 | 1.98 | 0.482 |
| diel_activityNocturnal | 0.36 | 0.64 | 0.56 | -0.89 | 1.60 | 0.576 |
| locationEurope | -1.16 | 1.22 | -0.95 | -3.55 | 1.23 | 0.342 |
| locationNorthAmerica | -1.89 | 1.22 | -1.55 | -4.28 | 0.50 | 0.121 |
| locationOceania | -1.38 | 1.20 | -1.15 | -3.73 | 0.97 | 0.251 |
| **Post-release short-term survival log-odds** | | | | | | |
| **Mammalia** | **Estimate** | **s.e.** | **Z-value** | **Lower** | **Upper** | **P-value** |
| **(I^2^ = 87.27) (AIC = 92.72)** |  |  |  |  |  |  |
| intercept | 0.39 | 1.76 | 0.22 | -3.07 | 3.84 | 0.826 |
| trophic_levelCarnivore | -0.92 | 1.69 | -0.55 | -4.23 | 2.39 | 0.585 |
| trophic_levelHerbivore | 0.37 | 1.53 | 0.24 | -2.63 | 3.37 | 0.811 |
| trophic_levelOmnivore | -0.01 | 1.49 | -0.01 | -2.93 | 2.90 | 0.992 |
| locationEurope | 0.56 | 1.37 | 0.41 | -2.11 | 3.24 | 0.680 |
| locationNorthAmerica | 0.10 | 1.43 | 0.07 | -2.70 | 2.91 | 0.943 |
| locationOceania | -0.02 | 1.38 | -0.01 | -2.72 | 2.69 | 0.991 |
| locationOutlier | 1.57 | 2.19 | 0.72 | -2.73 | 5.87 | 0.474 |
| **(I^2^ = 86.07) (AIC = 93.61)** |  |  |  |  |  |  |
| intercept | 1.90 | 2.48 | 0.76 | -2.97 | 6.76 | 0.445 |
| trophic_levelCarnivore | -0.13 | 1.55 | -0.09 | -3.18 | 2.91 | 0.931 |
| trophic_levelHerbivore | 0.05 | 1.37 | 0.04 | -2.64 | 2.73 | 0.972 |
| trophic_levelOmnivore | 0.47 | 1.41 | 0.33 | -2.29 | 3.23 | 0.740 |
| diel_activityCrepuscular | -0.93 | 2.23 | -0.42 | -5.30 | 3.43 | 0.676 |
| diel_activityDiurnal | -1.84 | 2.50 | -0.74 | -6.73 | 3.05 | 0.461 |
| diel_activityNocturnal | -1.80 | 2.26 | -0.80 | -6.22 | 2.62 | 0.425 |
| **Post-release long-term survival log-odds** | | | | | | |
| **Mammalia** | **Estimate** | **s.e.** | **Z-value** | **Lower** | **Upper** | **P-value** |
| intercept | -4.17 | 7.64 | -0.55 | -19.15 | 10.80 | 0.585 |
| trophic_levelCarnivore | 5.64 | 5.75 | 0.98 | -5.64 | 16.92 | 0.327 |
| trophic_levelHerbivore | 6.12 | 7.18 | 0.85 | -7.96 | 20.20 | 0.394 |
| trophic_levelOmnivore | 7.36 | 4.76 | 1.55 | -1.98 | 16.70 | 0.122 |
| diel_activityCrepuscular | -3.76 | 7.29 | -0.52 | -18.05 | 10.54 | 0.607 |
| diel_activityDiurnal | -6.13 | 10.62 | -0.58 | -26.94 | 14.69 | 0.564 |
| diel_activityNocturnal | -4.14 | 8.35 | -0.50 | -20.51 | 12.22 | 0.620 |
| locationEurope | 3.15 | 5.55 | 0.57 | -7.73 | 14.03 | 0.571 |
| locationNorthAmerica | 2.23 | 3.23 | 0.69 | -4.11 | 8.57 | 0.491 |
| adult_weight_classMedium | 3.56 | 7.00 | 0.51 | -10.17 | 17.29 | 0.612 |
| adult_weight_classSmall | -3.22 | 8.33 | -0.39 | -19.55 | 13.12 | 0.699 |
