# Supplementary material for: A systematic review of factors affecting wildlife survival during rehabilitation and release
Source: PLoS One. 2022 Mar 17;17(3):e0265514. doi: 10.1371/journal.pone.0265514 (PMC8929655; doi:10.1371/journal.pone.0265514)
Supplement: S4 Table — (DOCX) [file pone.0265514.s005.docx]

**S4 Table. A summary of reviewed articles and the survival measures reported for mammals and birds during care, and in the short- and long- term post release.**

| **Reference** | **Animal common name** | **Sample size (rehab)** | **Sample size (post-release)** | **Control group** | **Country** | **Unassisted death in care (%)** | **Survival at end of care (%)** | **Short-term survival post-release (%)** | **Long-term survival post-release (%)** | **Control survival post-release** | **Post-release survival measure** |
| --- | --- | --- | --- | --- | --- | --- | --- | --- | --- | --- | --- |
| **Aves** |  |  |  |  |  |  |  |  |  |  |  |
| **[1]** | Birds | 3597 | - | - | UK | - | 22 | - | - | - | - |
| **[2]** | Birds | 2390 | - | - | Spain | 27 | 57 | - | - | - | - |
| **Accipitriformes** |  |  |  |  |  |  |  |  |  |  |  |
| **[3]*^a^** | African white-backed vulture (*Gyps africanus*) | 132 | - | - | South Africa | - | 27.27 | - | - | - | - |
| **[4]*** | “^b^ | 38 | - | - | South Africa | - | 26.3 | - | - | - | - |
| **[5]** | Bald eagle (*Haliaeetus leucocephalus*) | - | 19 | - | USA | - | - | 68.4 | - | - | KTBA^c^ |
| **[6]** | Bald eagles (*Haliaeetus leucocephalus*) and peregrine falcons (*Falco peregrinus*) | 111 | - | - | USA | 20 | 52 | - | - | - | - |
| **[7]** | Cape Griffon vulture (*Gyps coprotheres*) |  | 163 | 242 | South Africa | - | - | - | 74.8 | 91.3 | ASR^d^ |
| **[4]*** | “ | 162 | - | - | South Africa | - | 38.9 | - | - | - | - |
| **[3]*** | “ | 560 | - | - | South Africa | - | 47.5 | - | - | - | - |
| **[8]*** | Common Buzzard (*Buteo buteo*) | 42 | - | - | Hungary | 28.60 | 31 | - | - | - | - |
| **[9]** | Eurasian Sparrowhawk (*Accipiter nisus*) | 205 | - | - | UK | 21 | 24 | - | - | - | - |
| **[10]** | Hawks | - | 9 | - | USA | - | - | 55 | - | - | KTBA |
| **[11]** | Raptors | 138 | - | - | Jordan | 16.00 | 84 | - | - | - | - |
| **[12]** | “ | 390 |  | - | USA | - | 25 | - | - | - | - |
| **[13]** | “ | 1693 | 648 | - | USA | - | 63.28 | - | - | - | - |
| **[14]** | “ | 1225 | - | - | USA | 19 | 42 | - | - | - | - |
| **[15]** | “ | 402 | - | - | Greece | 12.90 | 86.9 | - | - | - | - |
| **[16]** | “ | 242 | - | - | South Africa | 17 | 52 | - | - | - | - |
| **[17]** | “ | 6221 | - | - | Spain | 19.10 | 50.2 | - | - | - | - |
| **[18]** | “ | 2458 | - | - | Spain | 22.20 | 58.03 | - | - | - | - |
| **[19]** | “ | 2611 | - | - | Spain | 0.00 | 44.4 | - | - | - | - |
| **[20]** | “ | 154 | - | - | South Africa | 13 | 45 | - | - | - | - |
| **[21]** | “ | 484 | - | - | Australia | - | 53 | - | - | - | - |
| **[22]** | “ | 60 | - | - | USA | 26 | 59 | - | - | - | - |
| **Anseriformes** |  |  |  |  |  |  |  |  |  |  |  |
| **[23]** | Mallard duck (*Anas platyrhyncho*) | 1279 | - | - | British Columbia | 35 | 64.8 | - | - | - | - |
| **[24]*** | Surf scoter (*Melanitta perspicillata*) | - | 18 | 22 | USA | - | - | 14.3 | - | 49.8 | SR ^e^ |
| **[24] *** | “ | - | 15 | 22 | USA | - | - | 77.2 | - | 49.8 | SR |
| **Charadriiformes** |  |  |  |  |  |  |  |  |  |  |  |
| **[25]** | Great Knots (*Calidris tenuirostris*), Red Knots (*C. canutus),* Bar-tailed Godwits (*Limosa lapponica*), and Red-necked Stints (*C. ruficollis*) | 15 | 12 | 130 | Australia | 7 | 80 | 50 | - | - | KTBA |
| **[26]** | Common murre / guillemot (*Uria aalge*) | - | 1723 | - | Netherlands | - | - | - | 22 | - | SR |
| **[27] *** | “ | 913 | - | - | USA | 36.40 | 33.6 | - | - | - | - |
| **[27] *** | “ | 468 | - | - | USA | 15.80 | 27.6 | - | - | - | - |
| **[28]** | “ | 3798 | - | - | UK | 16.70 | 33.7 | - | - | - | - |
| **[29]** | “ | 550 | 31 | 25 | USA | - | - | 45 |  | 92 | KTBA |
| **[30]** | “ | - | 118 | 641 | USA | - | - | 12.6 | - | - | SR |
| **[31]** | “ | - | 309 | - | UK | - | - | 17 | 0.6 | 73 | ASR |
| **[32]** | Hooded Plover (*Thinornis rubricollis*) | 2 | 2 | - | Australia | 0 | 100 | - | 100 | - | NDR ^f^ |
| **[33]** | Western gull (*Larus occidentalis*) | 7 | 7 | 10 | USA | 0 | 100 | 100 | - | 90 | KTBA |
| **Columbiformes** |  |  |  |  |  |  |  |  |  |  |  |
| **[34]** | New Zealand Pigeon (*Hemiphaga novaeseelandiae*) | 76 | - | - | New Zealand | 5.48 | 52.63 | - | - | - | - |
| **[35]** | Woodpigeon (*Columba palumbus*) | 2653 | 13 | - | UK | - | 22.5 | 76.9 | 46.1 | - | KTBA |
| **Falconiformes** |  |  |  |  |  |  |  |  |  |  |  |
| **[36]** | Common Kestrel (*Falco tinnunculus*) | 108 | - | - | Hungary | 23.10 | 42.6 | - | - | - | - |
| **[37]** | Peregrine falcon (*Falco peregrinus*) | 161 | 66 |  | USA | - | 55 | 20 | 14 | 10-11 | SR |
| **Gruiformes** |  |  |  |  |  |  |  |  |  |  |  |
| **[38]** | American coot (*Fulica americana*) | 78 | 37 | 38 | USA | - | 60 | - | - | 76 | KTBA |
| **Passeriformes** |  |  |  |  |  |  |  |  |  |  |  |
| **[39]** | American robin (*Turdus migratorius*) | 688 | - | - | USA | - | 47 | - | - | - | - |
| **Pelecaniformes** |  |  |  |  |  |  |  |  |  |  |  |
| **[40]** | California brown pelican (*Pelecanus occidentalis californicus*) | - | 111 | 19 | USA | - | - | - | 0.09 | 53 | KTBA |
| **Psittaciformes** |  |  |  |  |  |  |  |  |  |  |  |
| **[41]** | Black cockatoo (*Calyptorhynchus spp.)* | 565 | NA | - | Australia | 10 | 43 | - | - | - | - |
| **[42]** | Carnaby's cockatoos (*Zanda latirostris*) | 760 | 23 | - | Australia | - | 19.1 | - | 73 | - | ASR |
| **Sphenisciformes** |  |  |  |  |  |  |  |  |  |  |  |
| **[43]** | African Penguin (*Spheniscus demersus*) | 3657 | - | - | South Africa | 9.82 | 83.3 | - | - | - | - |
| **[44]** | “ | 1772 | - | - | South Africa |  | 86 | - | - | - | - |
| **[45]** | “ | 1322 | 1117 | - | South Africa | 15.51 | 84 | - | 32 | - | ASR |
| **[46]** | “ | 10000 | 5213 | - | South Africa |  | 46 | 65 | 52 | - | KTBA |
| **[47]** | Little penguin (*Eudyptula minor*) | 1885 | 1788 | 1163 | Australia | 5 | 95 | - | 53 | - | KTBA |
| **[48]** | “ | 142 | NA | - | Australia | 61.27 | 39 | - | - | - | - |
| **Strigiformes** |  |  |  |  |  |  |  |  |  |  |  |
| **[49]** | Long-eared Owl (*Asio otus*) | 402 | - | - | Italy | - | 48.77 | - | - | - | - |
| **[8] *** | “ | 35 | - | - | Hungary | 20 | 37.1 | - | - | - | - |
| **[50]** | Tawny owl (*Strix aluco*) | - | 4 | - | UK | - | - | 75 | - | - | KTBA |
| **[51]** | “ | - | 57 | - | UK | - | - | 86 | - | - | SR |
| **[52]** | “ | - | 16 | - | UK | - | 70 | 46.2 | 33 | - | KTBA |
| **[53] *** | Western screech owl (*Otus kennecotti*) | - | 7 | - | USA | - | - | 62.5 | - | - | KTBA |
| **[53] *** | “ | - | 9 | - | USA | - | - | 50 | - | - | KTBA |
| **Suliformes** |  |  |  |  |  |  |  |  |  |  |  |
| **[54] *** | Cape gannet (*Morus capensis*) | 1297 | 138 | 5726 | South Africa | - | 65 | - | 85.5 | 88 | ASR |
| **[54] *** | “ | 194 | 24 | 4832 | South Africa | - | 65 | - | 88 | 90 | ASR |
| **Mammalia** |  |  |  |  |  |  |  |  |  |  |  |
| **[55]** | Badgers (*Meles meles*), blackbirds (*Turdus merula*), hedgehogs, red foxes (*Vulpes vulpes*), tawny owls (*Strix aluco*), starlings (*Sturnus vulgaris*) and house sparrows (*Passer domesticus*) | 5187 | - | - | UK | - | 39 | - | - | - | - |
| **Artiodactyla** |  |  |  |  |  |  |  |  |  |  |  |
| **[56]** | White-tailed deer (*Odocoileus virginianus*) | - | 41 | - | USA | - | - | 23.2 | - | - | SR |
| **[57]** | “ | - | 29 | - | USA | - | - | 14 | - | - | KTBA |
| **Carnivora** |  |  |  |  |  |  |  |  |  |  |  |
| **[58] *** | American black bear (*Ursus americanus*) | - | 424 | - | USA, Canada, Romania, Greece, South Korea and India | - | - | 73.4 | - | - | ASR |
| **[59]** | “ | - | 42 | - | USA | - | - | - | 93 | 73.4 | ASR |
| **[60]** | “ | - | 11 | - | USA | - | - | 77 | - | - | KTBA |
| **[61]** | “ | - | 52 | - | USA | - | - | - | 84.6 | - | KTBA |
| **[62]** | “ | - | 11 | - | USA | - | - | 100 | 60 | - | KTBA |
| **[63]** | Asiatic black bear (*Ursus thibetanus*) | - | 5 | - | India | - | - | - | 40 | - | - |
| **[58] *** | “ | - | 62 | - | USA, Canada, Romania, Greece, South Korea and India | - | - | 86.7 | - | - | ASR |
| **[58] *** | Brown bear (*Ursus arctos*) | - | 64 | - | USA, Canada, Romania, Greece, South Korea and India | - | - | 74.9 | - | - | ASR |
| **[64]** | Cheetah (*Acinonyx juabtus*) | - | 3 | - | Botswana | - | - | 0 | - | - | KTBA |
| **[65]** | Cheetah and leopard (*Panthera pardus*) | 4 | 4 | - | Botswana | 0 | 100 |  | 25 |  | KTBA |
| **[66]** | Grey seal (*Halichoerus grypus*) | 188 | - | - | UK | - | 81 | - | - | - | - |
| **[67]** | Harbor seal (*Phoca vitulina*) | 64 | - | - | USA | 30 | 66 | - | - | - | - |
| **[68]** | “ | 182 | - | - | British Columbia | 20.29 | 49.45 | - | - | - | - |
| **[69]** | Polecat (*Mustela putorius*) | 137 | 32 | - | UK | 8.33 | 78.57 | 50 | - | - | KTBA |
| **[70] *** | Raccoon dog (*Nyctereutes procyonoides*) | 68 | - | - | Japan | 57.40 | 42.6 | - | - | - | - |
| **[70] *** | “ | 157 |  | - | Japan | 38.90 | 61.1 | - | - | - | - |
| **[71]** | Sea otter (*Enhydra lutris*) | 357 | 45 | - | USA |  | 55.18 | - | 75.55 |  | KTBA |
| **[72] *** | “ | 26 | - | 12 | USA | - | - | - | 31 | 75 | KTBA |
| **[72] *** | “ | 7 | - | 12 | USA | - | - | - | 71 | 75 | KTBA |
| **[73]** | Stone marten (*Martes foina*) | - | 4 | - | Luxembourg | - | 100 | 75 | - | - | KTBA |
| **[74]** | Wolf (*Canis lupus*) | - | 2 | - | Portugal | - | 100 | 50 | 0 | - | KTBA |
| **Chiroptera** |  |  |  |  |  |  |  |  |  |  |  |
| **[75]** | Common and soprano pipistrelle bats (*Pipistrellus pipistrellus* and *P. pygmaeus*) | - | 10 | - | UK | - | - | 60 | - | - | KTBA |
| **[76]** | Flying fox (*Pteropus spp.)* | 22344 | - |  | Australia | 17.88 | 66 |  | - | - |  |
| **[77]** | Kuhl’s pipistrelle (*Pipistrellus kuhlii*) | - | 21 | - | Italy | - | - | 61.9 | - | - | KTBA |
| **Diprotodontia** |  |  |  |  |  |  |  |  |  |  |  |
| **[78]** | Common brushtail possum (*Trichosurus vulpecular*) | - | 20 | - | Australia | - | - | 40 | - | - | KTBA |
| **[79] *** | “ | - | 13 | - | Australia | - | - | 23.08 | - | - | KTBA |
| **[80]** | Common ringtail possum (*Pseudocheirus peregrinus*) | - | 112 | 41 | Australia | - | - | - | 0 | - | SR |
| **[81]** | “ | - | 22 | - | Australia | - | - | 9.09 | - | - | KTBA |
| **[82]** | Common wombat (*Vombatus ursinus*) | - | 13 | - | Australia | - | - | - | 92 | - | KTBA |
| **[83]** | “ | 54 | 44 | - | Australia | - | 82 | - | 77.3 | - | KTBA |
| **[84]** | Eastern grey kangaroo (*Macropus giganteus*) | - | 20 | - | Australia | - | - | - | 100 | - | NDR |
| **[85]** | “ | - | 87 | - | Australia | - | - | 97 | - | - | KTBA |
| **[86]** | Koala (*Phascolarctos cinereus*) | 10139 | - | - | Australia | 26 | 27 | - | - | - | - |
| **[87]** | “ | 12543 | - | - | Australia | 8.38 | 44 | - | - | - | - |
| **[88]** | “ | - | 4 | - | Australia | - | - | 100 | - | - | KTBA |
| **[89]** | “ | - | 12 | - | Australia | - | - | - | 70 | - | KTBA |
| **[90]** | “ | 88 | 46 | - | Australia | 3 | 52 | - | 50 | - | KTBA |
| **[91]** | “ | 17 | 16 | 23 | Australia | 6 | 94 | - | 58 | - | ASR |
| **[79] *** | “ | - | 27 | - | Australia | - | - | - | 77.78 | - | KTBA |
| **[92]** | Northern brush tailed possum (*Trichosurus arnhemensis*) | - | 42 | - | Australia | - | - | 69 | - | - | KTBA |
| **[93]** | Marsupials | 4608 | - | - | Australia | - | 85 | - | - | - | - |
| **Eulipotyphla** |  |  |  |  |  |  |  |  |  |  |  |
| **[94]** | European hedgehog (*Erinaceus europaeus*) | 740 | - | - | Portugal | 27.97 | 66.6 | - | - | - | - |
| **[95]** | “ | 490 | - | - | Spain | 28 | 69 | - | - | - | - |
| **[96]** | “ | - | 20 | 20 | UK | - | - | 73.1 |  | 63.6 | ASR |
| **[97]** | “ | - | 13 |  | UK | - | - | 76.92 | - | - | KTBA |
| **[98]** | “ | - | 8 | 3 | UK | - | - | 37.5 | - | - | KTBA |
| **[99]** | “ | - | 12 |  | UK | - | - | 33 | - | - | KTBA |
| **[100]** | “ | - | 34 | 23 | UK | - | - | 96.1 | 25.7 | 66 | SR |
| **[101]** | “ | 15 | 8 | 10 | Europe | - | 53.33 | 57.0 | - | 50 | KTBA |
| **Lagomorpha** |  |  |  |  |  |  |  |  |  |  |  |
| **[102]** | Desert and eastern cottontail (*Sylvilagus audubonii* and *S. floridanus*) | 150 | - | - | USA | - | 57 | - | - | - | - |
| **[103]** | Eastern cottontail rabbit (*Sylvilagus floridanus*) | 18709 | - | - | USA | 20 | 23 | - | - | - | - |
| **Perissodactyla** |  |  |  |  |  |  |  |  |  |  |  |
| **[104]** | Greater one-horned rhinoceros (*Rhinoceros unicornis*) | 3 | 3 | - | India | 0 | 100 | - | 100 | - | KTBA |
| **[105]** | White rhinoceros (*Ceratotherium simum*) | 3 | 3 | - | South Africa | 0 | 100 | 100 | - | - | KTBA |
| **Pholidota** |  |  |  |  |  |  |  |  |  |  |  |
| **[106]** | Temminck's pangolin (*Smutsia temminckii*) | - | 41 | - | South Africa | - | - | - | 70.73 | - | KTBA |
| **Primates** |  |  |  |  |  |  |  |  |  |  |  |
| **[107]** | African green monkey (*Chlorocebus aethiops*) | - | 29 | - | South Africa | - | - | 62 |  | 78 | KTBA |
| **[108]** | “ | - | 16 | - | South Africa | - | - | - | 37.5 | - | KTBA |
| **[109] *** | “ | - | 24 | - | South Africa | - | - | - | 74 | - | ASR |
| **[109] *** | “ | - | 35 | - | South Africa | - | - | - | 69 | - | ASR |
| **[110]** | Brown capuchin monkey (*Cebus apella*) | - | 8 | - | Colombia | - | - | 87.5 | - | - | KTBA |
| **Aves and Mammalia combined** |  |  |  |  |  |  |  |  |  |  |  |
| **[111]** | Birds and mammals | 145 | - | - | Turkey | 11.03 | 95.17 | - | - | - | - |
| **[112]** | Birds and mammals | 13375 | - | - | USA | - | 45.7 | - | - | - | - |

^a^ Asterisks indicate articles that present a survival measure for more than one study group

^b^ Quotation mark indicates the same species as listed above.

^c^ KTBA = known to be alive (a minimum survival) e.g. by trapping, observing or tracking with radio collars

^d^ ASR = annual survival rate e.g. using Kaplan-Meier calculation to estimate yearly survival

^e^ SR = survival rate e.g. using Kaplan-Meier calculation to estimate survival at a timepoint after release

^f^ NDR = no deaths recorded e.g. no mortality signals from radio collars

1. Baker, P.J., R. Thompson, and A. Grogan, *Survival rates of cat-attacked birds admitted to RSPCA wildlife centres in the UK: Implications for cat owners and wildlife rehabilitators.* Animal Welfare, 2018. **27**(4): p. 305-318.

2. Montesdeoca, N., et al., *Causes of morbidity and mortality, and rehabilitation outcomes of birds in Gran Canaria Island, Spain.* Bird Study, 2017. **64**(4): p. 523-534.

3. Howard, A., et al., *Injuries, mortality rates, and release rates of endangered vultures admitted to a rehabilitation center in South Africa.* Journal of Wildlife Rehabilitation, 2020. **40**(3): p. 15-24.

4. Naidoo, V., et al., *Vulture rescue and rehabilitation in South Africa: an urban perspective.* Journal of the South African Veterinary Association, 2011. **82**(1): p. 24-31.

5. Martell, M., et al., *Survival and movements of released rehabilitated bald eagles.* Biological Conservation, 1992. **62**(3): p. 231.

6. Harris, M.C. and M.S. Jonathan, *Morbidity and mortality of bald eagles (Haliaeetus leucocephalus) and peregrine falcons (Falco peregrinus) admitted to the Wildlife Center of Virginia, 1993–2003.* Journal of Zoo and Wildlife Medicine, 2007. **38**(1): p. 62-66.

7. Monadjem, A., et al., *Effect of rehabilitation on survival rates of endangered Cape vultures.* Animal Conservation, 2014. **17**(1): p. 52-60.

8. Sós-Koroknai, V., et al., *Causes of morbidity of common buzzards (Buteo buteo) and long-eared owls (Asio otus) at a Hungarian wildlife rescue centre between 2014 and 2016.* Biharean Biologist, 2020. **14**(1): p. 25-29.

9. Kelly, A. and M. Bland, *Admissions, diagnoses, and outcomes for Eurasian Sparrowhawks (Accipiter nisus) brought to a wildlife rehabilitation center in England.* Journal of Raptor Research, 2006. **40**(3): p. 231-235.

10. Hamilton, L.L., P.J. Zwank, and G.H. Olsen, *Movements and survival of released rehabilitated hawks.* Raptor Research, 1988. **22**(1): p. 22-26.

11. Al Zoubi, M.Y., et al., *Causes of raptor admissions to rehabilitation in Jordan.* Journal of Raptor Research, 2020. **54**(3): p. 273-278.

12. Deem, S.L., S.P. Terrell, and D.J. Forrester, *A retrospective study of morbidity and mortality of raptors in Florida: 1988-1994.* Journal of Zoo and Wildlife Medicine, 1998: p. 160-164.

13. Duke, G., P. Redig, and W. Jones, *Recoveries and resightings of released rehabilitated raptors.* Journal of Raptor Research, 1981. **15**(4): p. 97-107.

14. Hernandez, C.L., S.C. Oster, and J.L. Newbrey, *Retrospective study of raptors treated at the Southeastern Raptor Center in Auburn, Alabama.* Journal of Raptor Research, 2018. **52**(3): p. 379-388.

15. Komnenou, A.T., et al., *A retrospective study of presentation, treatment, and outcome of free-ranging raptors in Greece (1997–2000).* Journal of Zoo and Wildlife Medicine, 2005. **36**(2): p. 222-228.

16. Maphalala, M.I., et al., *Causes of admission to a raptor rehabilitation centre and factors that can be used to predict the likelihood of release.* African Journal of Ecology, 2021. **00**: p. 1-8.

17. Molina-López, R.A., J. Casal, and L. Darwich, *Final disposition and quality auditing of the rehabilitation process in wild raptors admitted to a wildlife rehabilitation centre in Catalonia, Spain, during a twelve year period (1995-2007).* PLoS ONE, 2013. **8**(4).

18. Montesdeoca, N., et al., *Final outcome of raptors admitted to the Tafira wildlife rehabilitation center, gran Canaria island, Spain (2003-2013).* Animal Biodiversity and Conservation, 2017. **40**(2): p. 211-220.

19. Rodríguez, B., et al., *Causes of raptor admissions to a wildlife rehabilitation center in Tenerife (Canary Islands).* Journal of Raptor Research, 2010. **44**(1): p. 30-39.

20. Thompson, L.J., B. Hoffman, and M. Brown, *Causes of admissions to a raptor rehabilitation centre in KwaZulu-Natal, South Africa.* African Zoology, 2013. **48**(2): p. 359-366.

21. Thomson, V.K., et al., *Hospital Admissions of Australian Coastal Raptors Show Fishing Equipment Entanglement is an Important Threat.* Journal of Raptor Research, 2020. **54**(4): p. 414-423.

22. Fix, A.S. and S.Z. Barrows, *Raptors rehabilitated in Iowa during 1986 and 1987: a retrospective study.* Journal of Wildlife Diseases, 1990. **26**(1): p. 18-21.

23. Drake, A., *Mallard duckling care and survival at a wildlife rehabilitation center*. 2007, University of British Columbia.

24. De La Cruz, S.E.W., et al., *Post-release survival of surf scoters following an oil spill: An experimental approach to evaluating rehabilitation success.* Marine Pollution Bulletin, 2013. **67**(1-2): p. 100-106.

25. Rogers, D.I., et al., *Treatment of capture myopathy in shorebirds: a successful trial in northwestern Australia.* Journal of Field Ornithology, 2004. **75**(2): p. 157-164.

26. Camphuysen, C., et al., *Recoveries of guillemots ringed in the Netherlands: the survival of rehabilitated oiled seabirds.* SULA, 1997. **11**(3): p. 157-174.

27. Duerr, R.S., M.H. Ziccardi, and J. Gregory Massey, *Mortality during treatment: Factors affecting the survival of oiled, rehabilitated common murres (Uria aalge).* Journal of Wildlife Diseases, 2016. **52**(3): p. 495-505.

28. Grogan, A., et al. *Factors affecting the welfare and rehabilitation of oiled murres (Uria aalge) in England and Wales, UK*. in *The International Wildlife Rehabilitation Council symposium*. 2011. Coral Springs, FL.

29. Newman, S.H., et al., *The effects of petroleum exposure and rehabilitation on post-release survival, behavior, and blood health indices: A common murre (Uria aalge) case study following the Stuyvesant petroleum spill.* Final Report. Oiled Wildlife Care Network, Wildlife Health Center, UC Davis, CA, 2004.

30. Sharp, B.E., *Post-release survival of oiled, cleaned seabirds in North America.* Ibis, 1996. **138**(2): p. 222-228.

31. Wernham, C., W.J. Peach, and S.J. Browne, *Survival rates of rehabilitated guillemots*. 1997: British Trust for Ornithology Thetford, Norfolk.

32. Weston, M.A., et al., *Can oiled shorebirds and their nests and eggs be successfully rehabilitated? A case study involving the threatened hooded plover Thinornis rubricollis in south-eastern Australia.* Waterbirds, 2008. **31**(1): p. 127-132.

33. Golightly, R.T., et al., *Survival and behavior of western gulls following exposure to oil and rehabilitation.* Wildlife Society Bulletin, 2002. **30**(2): p. 539-546.

34. Cousins, R.A., P.F. Battley, and B.F. Gartrell, *Impact injuries and probability of survival in a large semiurban endemic pigeon in New Zealand, Hemiphaga novaeseelandiae.* Journal of Avian Medicine and Surgery, 2012. **26**(4): p. 274.

35. Kelly, A., et al., *Factors affecting the likelihood of release of injured and orphaned woodpigeons (Columba palumbus).* Animal Welfare, 2011. **20**(4): p. 523-534.

36. Sós-Koroknai, V., et al., *Examination of morbidity and the incidence of electrocution in common kestrels (Falco tinnunculus) admitted to the Wildlife Rescue Centre at the Budapest Zoo and Botanical Garden between 2014 and 2016.* Magyar Allatorvosok Lapja, 2020. **142**(7): p. 429-438.

37. Sweeney, S.J., P.T. Redig, and H.B. Tordoff, *Morbidity, survival and productivity of rehabilitated Peregrine Falcons in the upper midwestern U.S.* Journal of Raptor Research, 1997. **31**(4): p. 347-352.

38. Anderson, D.W., et al., *An experimental soft-release of oil-spill rehabilitated American coots (Fulica americana): I. Lingering effects on survival, condition and behavior.* Environmental Pollution (1987), 2000. **107**(3): p. 285-294.

39. Haynes, E., H.N. Erb, and J. Nevis, *Statistical analysis of juvenile American robin rehabilitation at Willowbrook Wildlife Center, Illinois, USA: can admission weight be used to predict rehabilitation outcome?* Journal of Wildlife Rehabilitation, 2013. **33**(1).

40. Anderson, D.W., F. Gress, and D.M. Fry, *Survival and dispersal of oiled brown pelicans after rehabilitation and release.* Marine Pollution Bulletin, 1996. **32**(10): p. 711-718.

41. Le Souëf, A., et al., *Presentation and prognostic indicators for free-living black cockatoos (Calyptorhynchus Spp.) admitted to an Australian Zoo Veterinary Hospital over 10 years.* Journal of Wildlife Diseases, 2015. **51**(2): p. 380-388.

42. Groom, C.J., K. Warren, and P.R. Mawson, *Survival and reintegration of rehabilitated Carnaby's cockatoos Zanda latirostris into wild flocks.* Bird Conservation International, 2018. **28**(1): p. 86.

43. Parsons, N.J., R.E. Vanstreels, and A.M. Schaefer, *Prognostic indicators of rehabilitation outcomes for adult African penguins (Spheniscus demersus).* Journal of Wildlife Diseases, 2018. **54**(1): p. 54-65.

44. Parsons, N. and L. Underhill, *Oiled and injured African penguins Spheniscus demersus and other seabirds admitted for rehabilitation in the Western Cape, South Africa, 2001 and 2002.* African Journal of Marine Science, 2005. **27**(1): p. 289-296.

45. Sherley, R.B., et al., *Hand-rearing, release and survival of African penguin chicks abandoned before independence by moulting parents.* PloS ONE, 2014. **9**(10): p. e110794.

46. Underhill, L.G., et al., *Mortality and survival of African penguins Spheniscus demersus involved in the Apollo Seaoil spill: an evaluation of rehabilitation efforts.* Ibis, 1999. **141**(1): p. 29-37.

47. Goldsworthy, S.D., et al., *Effects of the Iron Baron oil spill on little penguins (Eudyptula minor). II. Post-release survival of rehabilitated oiled birds.* Wildlife Research, 2000. **27**(6): p. 573-582.

48. Jessop, R. and P. Du Guesclin, *The effects of an oil spill at Apollo Bay, Victoria, on little penguins Eudyptula minor in May 1990.* Australian Bird Watcher, 2000. **18**(5): p. 192-198.

49. Mariacher, A., et al., *Causes of admission and outcomes of long-eared owl (Asio otus) in wildlife rescue centres in Italy from 2010 to 2014.* Avian Biology Research, 2016. **9**(4): p. 282-286.

50. Bennett, J. and A. Routh, *Post-release survival of hand-reared tawny owls (Strix aluco).* Animal Welfare, 2000. **9**(3): p. 317-321.

51. Griffiths, R., C. Murn, and R. Clubb, *Survivorship of rehabilitated juvenile tawny owls (Strix aluco) released without support food, a radio tracking study.* Avian Biology Research, 2010. **3**(1): p. 1-6.

52. Leighton, K., et al., *Post-release survival of hand-reared tawny owls (Strix aluco) based on radio-tracking and leg-band return data.* Animal Welfare, 2008. **17**(3): p. 207-214.

53. Allbritten, M. and D. Jackson, *A postrelease study of rehabilitated western screech owls (Otus kennecotti) in Douglas County, oregon.* Journal of Wildlife Rehabilitation, 2002. **25**(4): p. 5-10.

54. Altwegg, R., et al., *Long-term survival of de-oiled Cape gannets Morus capensis after the Castillo de Bellver oil spill of 1983.* Biological Conservation, 2008. **141**(7): p. 1924-1929.

55. Molony, S.E., et al., *Factors that can be used to predict release rates for wildlife casualties.* Animal Welfare, 2007. **16**(3): p. 361-367.

56. Beringer, J., et al., *Post-release survival of rehabilitated white-tailed deer fawns in Missouri.* Wildlife Society Bulletin, 2004. **32**(3): p. 732-738.

57. Williams, S.C. and M.A. Gregonis, *Survival and movement of rehabilitated white‐tailed deer fawns in Connecticut.* Wildlife Society Bulletin, 2015. **39**(3): p. 664-669.

58. Beecham, J.J., et al., *Management implications for releasing orphaned, captive-reared bears back to the wild.* The Journal of Wildlife Management, 2015. **79**(8): p. 1327-1336.

59. Blair, C.D., et al., *Survival and conflict behavior of American black bears after rehabilitation.* Journal of Wildlife Management, 2020. **84**(1): p. 75-84.

60. Clark, J.E., et al., *Survival of orphaned black bears released in the Smoky Mountains.* Ursus, 2002: p. 269-273.

61. Hashem, B.J., *Evaluating the success of an orphaned American black bear (Ursus americanus) rehabilitation program in Virginia.* Journal of Wildlife Rehabilitation, 2019. **39**(2): p. 7-12.

62. Smith, W.E., et al., *Short-term fate of rehabilitated orphan black bears released in New Hampshire.* Human–Wildlife Interactions, 2016. **10**(2): p. 14.

63. Ashraf, N., *Walking the Bears: Rehabilitation of Asiatic Black Bears in Arunachal Pradesh*. 2008: Wildlife Trust of India.

64. Houser, A., *Spoor density, movement and rehabilitation of cheetahs in Botswana*. 2009, University of Pretoria.

65. Houser, A., et al., *Pre-release hunting training and post-release monitoring are key components in the rehabilitation of orphaned large felids.* African Journal of Wildlife Research, 2011. **41**(1): p. 11-20.

66. Barnett, J. and S. Westcott, *Distribution, demographics and survivorship of grey seal pups (Halichoerus grypus) rehabilitated in southwest England.* Mammalia, 2001. **65**(3): p. 349-361.

67. Greig, D.J., et al., *Hematology and serum chemistry in stranded and wildcaught harbor seals in central California: Reference intervals, predictors of survival, and parameters affecting blood variables.* Journal of Wildlife Diseases, 2010. **46**(4): p. 1172-1184.

68. MacRae, A., M. Haulena, and D. Fraser, *The effect of diet and feeding level on survival and weight gain of hand‐raised harbor seal pups (Phoca vitulina).* Zoo Biology, 2011. **30**(5): p. 532-541.

69. Kelly, A., R. Scrivens, and A. Grogan, *Post-release survival of orphaned wild-born polecats Mustela putorius reared in captivity at a wildlife rehabilitation centre in England.* Endangered Species Research, 2010. **12**(2): p. 107-115.

70. Kido, N., et al., *Effective treatment for improving the survival rate of raccoon dogs infected with Sarcoptes scabiei.* Journal of Veterinary Medical Science, 2014. **76**(8): p. 1169-1172.

71. Estes, J.A. and M.T. Tinker, *Rehabilitating sea otters: Feeling good versus being effective*, in *Effective Conservation Science: Data Not Dogma*. 2017. p. 128-134.

72. Nicholson, T.E., et al., *Effects of rearing methods on survival of released free-ranging juvenile southern sea otters.* Biological Conservation, 2007. **138**(3): p. 313-320.

73. Herr, J., L. Schley, and T.J. Roper, *Fate of translocated wild-caught and captive-reared stone martens (Martes foina).* European Journal of Wildlife Research, 2008. **54**(3): p. 511-514.

74. Rio-Maior, H., et al., *Rehabilitation and post-release monitoring of two wolves with severe injuries.* Journal of Wildlife Management, 2016. **80**(4): p. 729-735.

75. Kelly, A., et al., *Further evidence for post-release survival of hand-reared, orphaned bats based on radio-tracking and ring-return data.* Animal Welfare-The UFAW Journal, 2012. **21**(1): p. 27.

76. Mo, M., et al., *Using wildlife carer records to identify patterns in flying-fox rescues: A case study in New South Wales, Australia.* Pacific Conservation Biology, 2020.

77. Serangeli, M., et al., *The post-release fate of hand-reared orphaned bats: survival and habitat selection.* Animal Welfare-The UFAW Journal, 2012. **21**(1): p. 9.

78. Herbert, C., et al. *Brushtail Possums in Care: Factors influencing post-release survival and the potential impacts of stress on release outcomes*. in *Australian WIldlife Rehabilitation Conference*. 2018. Sydney.

79. Tribe, A. *Measuring the success of wildlife rehabilitation*. in *National Wildlife Rehabilitation Conference, Surfers Paradise*. 2005. Gold Coast.

80. Augee, M.L., B. Smith, and S. Rose, *Survival of wild and hand-reared ringtail possums (Pseudocheirus peregrinus) in bushland near Sydney.* Wildlife Research, 1996. **23**(1): p. 99-108.

81. Russell, B., B. Smith, and M. Augee, *Changes to a population of common ringtail possums (Pseudocheirus peregrinus) after bushfire.* Wildlife Research, 2003. **30**(4): p. 389-396.

82. Ridgeway, P. *Rewilding ecosystems through wildlife rehabilitation – a successful trial in Western Sydney*. in *Australian Wildlife Rehabilitation Conference*. 2018. Sydney.

83. Saran, K., et al., *Rehabilitation as a conservation tool: a case study using the common wombat.* Pacific Conservation Biology, 2011. **17**(4): p. 310-319.

84. Campbell, L. and D. Croft. *Comparison of hard and soft release of hand reared eastern grey kangaroos*. in *Veterinary conservation biology, wildlife health and management in Australasia, proceedings of international joint conference. Sydney: Taronga Zoo*. 2001.

85. Garlick, S. and R. Austen. *Kangaroo translocation: program efficiency and welfare goals*. in *National Wildlife Rehabilitation Conference SA*. 2010. Adelaide.

86. Burton, E. and A. Tribe, *The rescue and rehabilitation of koalas (Phascolarctos cinereus) in southeast Queensland.* Animals, 2016. **6**(9).

87. Charalambous, R. and E. Narayan, *A 29-year retrospective analysis of koala rescues in New South Wales, Australia.* Plos One, 2020. **15**(10).

88. Ellis, W.A.H., et al., *Response of koalas (Phascolarctos cinereus) to re-introduction to the wild after rehabilitation.* Australian wildlife research, 1990. **17**(4): p. 421-426.

89. Goldingay, R.L. and B. Dobner, *Home range areas of koalas in an urban area of north-east New South Wales.* Australian Mammalogy, 2014. **36**(1): p. 74-80.

90. Griffith, J.E. and D.P. Higgins, *Diagnosis, treatment and outcomes for koala chlamydiosis at a rehabilitation facility (1995-2005).* Australian Veterinary Journal, 2012. **90**(11): p. 457-463.

91. Lunney, D., et al., *Post-fire survival and reproduction of rehabilitated and unburnt koalas.* Biological Conservation, 2004. **120**(4): p. 567-575.

92. Neyens, J. and S. Hirst. *Survival of released rehabilitated northern brush tailed possums (Trichosurus arnhemensis)*. in *Australian Wildlife Rehabilitation Conference*. 2014. Hobart.

93. Englefield, B., et al., *The Demography and Practice of Australians Caring for Native Wildlife and the Psychological, Physical and Financial Effects of Rescue, Rehabilitation and Release of Wildlife on the Welfare of Carers.* Animals, 2019. **9**(12).

94. Garcês, A., et al., *Outcomes, mortality causes, and pathological findings in european hedgehogs (Erinaceus europeus, Linnaeus 1758): A seventeen year retrospective analysis in the North of Portugal.* Animals, 2020. **10**(8): p. 1-13.

95. Martínez, J.C., A.I. Rosique, and M.S. Royo, *Causes of admission and final dispositions of hedgehogs admitted to three wildlife rehabilitation centers in eastern Spain.* Hystrix, 2014. **25**(2): p. 107-110.

96. Molony, S.E., et al., *The effect of translocation and temporary captivity on wildlife rehabilitation success: An experimental study using European hedgehogs (Erinaceus europaeus).* Biological Conservation, 2006. **130**(4): p. 530-537.

97. Morris, P.A., *Released, rehabilitated hedgehogs: A follow-up study in Jersey.* Animal Welfare, 1997. **6**(4): p. 317-327.

98. Morris, P., K. Meakin, and S. Sharafi, *The behaviour and survival of rehabilitated hedgehogs (Erinaceus europaeus).* Animal Welfare, 1993. **2**(1): p. 53-66.

99. Morris, P.A. and H. Warwick, *A Study of Rehabilitated Juvenile Hedgehogs After Release into the Wild.* Animal Welfare, 1994. **3**(3): p. 163-177.

100. Yarnell, R.W., et al., *Should rehabilitated hedgehogs be released in winter? A comparison of survival, nest use and weight change in wild and rescued animals.* European Journal of Wildlife Research, 2019. **65**(1).

101. Rasmussen, S.L., et al., *An exploratory investigation of glucocorticoids, personality and survival rates in wild and rehabilitated hedgehogs (Erinaceus europaeus) in Denmark.* BMC Ecology and Evolution, 2021. **21**(1): p. 96.

102. Paul, G. and D.G. Friend, *Comparison of outcomes using two milk replacer formulas based on commercially available products in two species of infant cottontail rabbits.* Journal of Wildlife Rehabilitation, 2017. **37**(1).

103. Santos, A.R.B.M.F., *Eastern cottontail rabbit (Sylvilagus floridanus) admission causes and corresponding outcomes at the Wildlife Rehabilitation Center of Minnesota: a retrospective study from 2011 to 2017*. 2018, Universidade de Lisboa, Faculdade de Medicina Veterinária.

104. Barman, R., et al., *Rehabilitation of greater one-horned rhinoceros calves in Manas National Park, a World Heritage Site in India.* Journal of Wildlife Rehabilitation, 2019. **39**(1): p. 17-26.

105. Miazga, K., et al., *Releasing three orphaned white rhinoceroses (Ceratotherium simum) to the game reserve in South Africa. Rehabilitation, translocation and post-release observations.* Animals, 2020. **10**(12): p. 1-15.

106. Meyer, F.C., *Survival and distribution of Temminck’s pangolin (Smutsia temminckii) retrieved from the illegal wildlife trade in South Africa*. 2020, University of Venda.

107. Guy, A.J., *Release of rehabilitated Chlorocebus aethiops to Isishlengeni Game Farm in KwaZulu-Natal, South Africa.* Journal for Nature Conservation, 2013. **21**(4): p. 214-216.

108. Guy, A.J., O.M.L. Stone, and D. Curnoe, *Assessment of the release of rehabilitated vervet monkeys into the Ntendeka Wilderness Area, KwaZulu-Natal, South Africa: a case study.* Primates, 2012. **53**(2): p. 171-179.

109. Wimberger, K., C. Downs, and M. Perin, *Postrelease success of two rehabilitated vervet monkey (Chlorocebus aethiops) troops in KwaZulu-Natal, South Africa.* Folia Primatologica, 2010. **81**: p. 96–108.

110. Suarez, C., et al., *Survival and adaptation of a released group of confiscated capuchin monkeys.* Animal Welfare, 2001. **10**(2): p. 191-203.

111. Aslan, L., Ö. Adizel, and T. Sancak, *Treatment and rehabilitation of wild birds and mammals.* Indian Journal of Animal Research, 2018. **52**(4): p. 623-627.

112. Doell, D. and D.A. Locky, *Trends in wildlife intake at a rehabilitation center in Central Alberta: A retrospective analysis of birds, mammals, and herptiles, from 1990 through 2012.* Journal of Wildlife Rehabilitation, 2016. **36**(1): p. 17-29.
