# Supplementary material for: A systematic review of factors affecting wildlife survival during rehabilitation and release
Source: PLoS One. 2022 Mar 17;17(3):e0265514. doi: 10.1371/journal.pone.0265514 (PMC8929655; doi:10.1371/journal.pone.0265514)
Supplement: S5 Table — (DOCX) [file pone.0265514.s006.docx]

# Supplementary Table 5. A summary of factors associated with wildlife survival during rehabilitation and after release, with the direction of effects (higher or lower probability of survival), relevant species and references, and factors categorised into event-related, intrinsic or individual (inter-specific and intra-specific), intervention-related, release environment, and human-wildlife interface.

| **Factor** | **Phase affected** | **Higher probability of survival after rescue** | **Lower probability of survival after rescue** | **Species and Reference** |
| --- | --- | --- | --- | --- |
| **Event-related** | | | | |
| **Reason for admission and associated diagnosis and severity** | Survival to release | Less severe reason for rescue or diagnosis, e.g. uninjured orphans^a^ | More severe reason for rescue or diagnosis, e.g. fracture^a^ | Black cockatoos (*Calyptorhynchus* spp.) [1], flying foxes (*Pteropus* spp.) [2], little penguins (*Eudyptula minor)* [3, 4], birds [5-10], vultures (*Gyps* spp.) [11, 12], raptors [13-21], long-eared owls (*Asio otus*) [22], African penguins (*Spheniscus demersus*) [23], grey seals (*Halichoerus grypus*) [24], hedgehogs (*Erinaceus europaeus*) [25], cottontail rabbits (*Sylvilagus floridanus*) [26], badgers (*Meles meles*), blackbirds (*Turdus merula*), hedgehogs, red foxes (*Vulpes vulpes*), tawny owls (*Strix aluco*), starlings (*Sturnus vulgaris*) and house sparrows (*Passer domesticus*) [27] |
|  | Survival post-release | Less extensive oiling^a^ | More extensive oiling^a^ | Little penguins [3] |
| **Size of disaster** | Survival to release | Major oil spill event i.e. greater search and recovery effort^a^ [28] | Widespread events (e.g. heat stress) can overwhelm rehabilitator capacity^b^ [2] | Common murres (*Uria aalge*) [28] and flying foxes [2] |
| **Season of admission** | Survival to release | N/A^c^ | Coincides with physiologically taxing events, such as breeding or moulting^a^ | African penguins [23] |
| **Intrinsic or individual - inter-specific** | | | | |
| **Body size** | Survival to release | Larger body size^b^ | N/A | Cape gannet (*Morus capensis*), African penguin [29] |
| **Migratory or not** | Survival post-release | Non-migratory (i.e. no strain of migration soon after release)^b^ | Migratory (i.e. become oiled far from breeding localities)^b^ | Cape gannet, common murre [29] |
| **Behaviour** | Survival to release | Resilient and easily adapts to captivity^b^ | N/A | European hedgehogs [30] |
|  | Survival post-release | Easily adapts to post-release environment^b^ | N/A | Kuhl’s pipistrelle bats (*Pipistrellus kuhlii*) [31] |
| **Intrinsic or individual - intra-specific** | | | | |
| **Age** | Survival to release | Juveniles may present with less severe injuries such as orphaning, so have greater survival than adults^a^ [2, 5, 6, 13, 19, 22, 26, 32, 33] | Juveniles can have lower survival than adults^a^, often due to characteristics of their age such as moult, presence of an umbilicus (susceptible to infection) or differing fitness requirements [28, 34-36] | Flying foxes [2], wombats (*Vombatus ursinus*) [32], raptors [13, 19], owls [22], birds [5, 6, 28, 34], cottontail rabbits [26], harbour seals (*Phoca vitulina*) [35], and koalas (*Phascolarctos cinereus*) [33, 36] |
|  | Survival post-release | Juvenile pelicans had better survival than adults^a^ [37] | Juvenile (hand-reared) possums did not survive as long as adults^a^ [38] | Brushtail possums (*Trichosurus vulpecula*) [38], brown pelicans (*Pelecanus occidentalis californicus*) [37] |
| **Sex** | Survival to release | Male polecats were more likely to be released than females^a^ [39] | Male sparrowhawks were less likely to be released than females^a^ [20], male raptors were more susceptible to unassisted mortality than females [16] | Polecats (*Mustela putorius*) [39], sparrowhawks (*Accipiter nisus*) [20], raptors [16] |
|  | Survival post-release | Male little penguins had higher survival rates than females^a^ [3] | N/A | Little penguins [3] |
| **Bodyweight** | Survival to release | Heavier bodyweight at time of rescue^a^ | Lower rescue mass and poor rescue condition^a^ | Common murres [28], American robins (*Turdus migratorius*) [40], African penguins [23], harbour seals [35, 41], woodpigeons (*Columba palumbus*) [6], cottontail rabbits [26], and mallard ducklings (*Anas platyrhyncho*) [42] |
|  | Survival post-release | As above^a^ | As above^a^ | Little penguins [3] |
| **Individual personality** | Survival post-release | More exploratory individuals^a^ | Less exploratory individuals^a^ | Brushtail possums [43] |
| **Brood size** | Survival to release | Improved juvenile survival when larger brood is rescued^a^ | N/A | Mallard ducks [42] |
| **Physiological parameters** | Survival to release | Higher body temperature, higher packed cell volume and higher total plasma protein^a^ [10] | Low total plasma protein, low haematocrit^a^ [23] | Common murres [10], African penguins [23] |
| **Activity pattern** | Survival to release | Young diurnal raptors were admitted more frequently and had greater release rates than adults^a^ | Adult nocturnal raptors were admitted more frequently than young birds^a^. Diurnal birds were more often treated for fractures than nocturnal birds^a^ | Raptors [13] |
| **Intervention-related** | | | | |
| **Personnel and facilities for rescue, transport, care, and release** | Survival to release | Readily available and adequately equipped care facilities, trained personnel, and refined protocols^b^ | Birds delivered by the public to the wildlife care centre (versus an animal collection officer or veterinarian)^a^; time delay between event (e.g. oil spill) and rescue or veterinary treatment^b^ | Shorebirds [44, 45], western gulls (*Larus occidentalis*) [46], Cape gannet [29], common murres [28], African penguins [47], birds [5], little penguins [4], raptors [48] |
|  | Survival post-release | As above^b^ | N/A | Shorebirds [45], common murres [49] |
| **Wildlife care centre** | Survival to release | Some centres had higher survival^a^, possibly due to increased levels of experience and appropriate triage and treatment regimes | Some centres had lower survival^a^, possibly due to lower levels of experience and ineffective or inappropriate triage and treatment regimes | Koalas [50], birds [5] |
| **Choice of hand-raising diet** | Survival to release | Artificial milk-replacer had greater survival than fish-formula^a^, and a commercial milk-replacer was better than another commercial brand^a^ | N/A | Seals [41], cottontail rabbits [51] |
| **Maintenance of bodyweight** | Survival post-release | Sufficient fat reserves or heavier mass on release^ab^ | Weight loss in migratory birds^b^ | Shorebirds [44], hedgehogs [52, 53], American black bears [54, 55] |
| **Treatment regimens** | Survival to release | Individuals with a better response to treatment^a^; disease management and ancillary treatment^a^ | Treatment based on clinical signs rather than diagnostic tests^b^; incorrect drug dosages given by rehabilitators^b^, inappropriate treatments^b^ | Wombats [32], koalas [33], little penguins [4], and raccoon dogs (*Nyctereutes procyonoides*) [56] |
|  | Survival post-release | Appropriate disease management^b^ | Inadequate oil removal procedures^b^; failure to eliminate pathogen before release^b^ | Koalas [33, 57], marine birds [37, 58, 59] |
| **Habituation to humans** | Survival to release | Less human-imprinted animals are more likely to survive^a^ [32] | N/A | Wombats [32] |
|  | Survival post-release | Shorter periods of rehabilitation may be better^b^ [38], although duration did not affect little penguin survival^a^ [3] | Loss of wild behaviours such as predator avoidance and disruption of social development due to human habituation^ab^, although habituation was not related to survival in deer^a^ [60] | Possums [38, 43], little penguins [3], wombats [32], deer (*Odocoileus virginianus*) [60], cheetahs (*Acinonyx jubatus*) and leopards (*Panthera pardus*) [61], rhinoceros (*Ceratotherium simum*) [62], American black bear [55], sea otters (*Enhydra lutris*) [63] |
| **Hunting and wild behaviour training** | Survival to release | Construction of a pre-release flight tunnel for raptors^b^ [48] | N/A | Raptors [48] |
|  | Survival post-release | Provision of suitable hunting training^b^ | Lack of pre-release training to navigate situations likely to be encountered in the wild^b^; the mother-fawn relationship is essential, so hand-reared fawns lacked traits required for survival^b^ [64] | Cheetahs and leopards [61, 65], capuchin monkeys (*Cebus apella*) [66], pipistrelle bats [67], fawns [64] |
| **Formation of social groups in captivity** | Survival post-release | Formation of social groups and surrogate mentor females provided for juveniles^ab^. | Lack of group cohesiveness prior to release^b^. | Cockatoos [68], sea otters [63], African green monkeys (*Chlorocebus aethiops*) [69] |
| **Readiness for release** | Survival post-release | Yearling age improves survival in bear cubs^a^; adequate waterproofing and bouyancy for water birds^b^ | Released with unresolved ailments or long-term effects from reason for rescue^b^ | Bears [54, 55, 70], marine birds [9, 37, 49, 59], pangolins (*Smutsia temminckii*) [71] |
| **Release environment** | | | | |
| **Timing of release** | Survival post-release | Release during the non-breeding season^a^; mild weather^b^; high prey or food availability^b^ | Majority of hedgehog mortalities occurred during spring when they are most active^a^ [52] | Possums [38], hedgehogs [52], common murres [49], American black bears [70], western gulls [46] |
| **Release method** | Survival post-release | Release to a wild flock or known congregation area^b^; release of female macropods in groups with other female rearing companions^b^; soft release^b^ [64, 71, 72]^a^; release close to breeding locations^b^ | Hard released Asiatic black bears had shorter survival than soft-released bears^a^ [72] | Cockatoos [68], kangaroos (*Macropus giganteus*) [73], hedgehogs [52], rhinoceros (*Rhinoceros unicornis*) [74], bears [72], pangolin [71], pipistrelle bats [31], Cape gannets [29], deer [64] |
| **Familiarity of habitat** | Survival post-release | Familiar release habitat may not be necessary for all species [3, 38, 75]^b^ | Unfamiliar release habitat^b^ | Koalas [38], hedgehogs [75, 76], little penguins [3], possums [77, 78], deer [60] |
| **Habitat quality** | Survival post-release | Unfamiliar habitat may be suitable while there are sufficient food trees and the carrying capacity has not been exceeded^b^  [38, 79] | At or near carrying capacity^b^; reduced canopy continuity following bushfires^b^; competition and attacks from conspecifics^b^; the need to immediately travel long distances^b^; the presence of illegal hunting activity and proximity to dwellings and roads^b^. | Koala [38, 79], possums [78, 80], hawks [81], monkeys [66, 82, 83], bald eagles (*Haliaeetus leucocephalus*) [84], rhinoceros [62] |
| **Predators** | Survival post-release | Control or absence of predators^b^ | Presence of predators^b^ | Koalas [85], wombats [86], monkeys [66] |
| **Human-wildlife interface** | | | | |
| **Increasing human population and habitat fragmentation** | Survival post-release | N/A | Stress associated with bushfires and habitat fragmentation may be contributing to disease in koalas^b^ | Koalas [36, 87] |
| **Hunting activities** | Survival post-release | N/A | Survival rates of bears reflect their permitted hunting pressure^b^ | Asiatic black bears, American black and brown bears [54] |
| **Urban expansion** | Survival post-release | N/A | Increasing encounters in recolonised areas results in more illegal kills^b^ | Asiatic black bears, American black and brown bears [54] |

1. Le Souëf A, Holyoake C, Vitali S, Warren K. Presentation and prognostic indicators for free-living black cockatoos (*Calyptorhynchus* Spp.) admitted to an Australian Zoo Veterinary Hospital over 10 years. Journal of Wildlife Diseases. 2015;51(2):380-8. doi: 10.7589/2014-08-203.

2. Mo M, Roache M, Haering R, Kwok A. Using wildlife carer records to identify patterns in flying-fox rescues: A case study in New South Wales, Australia. Pacific Conservation Biology. 2020;27(1):61-9. doi: 10.1071/PC20031.

3. Goldsworthy SD, Giese M, Gales RP, Brothers N, Hamill J. Effects of the Iron Baron oil spill on little penguins (*Eudyptula minor*). II. Post-release survival of rehabilitated oiled birds. Wildlife Research. 2000;27(6):573-82. doi: 10.1071/wr99076. PubMed PMID: WOS:000165567500002.

4. Jessop R, Du Guesclin P. The effects of an oil spill at Apollo Bay, Victoria, on little penguins *Eudyptula minor* in May 1990. Australian Bird Watcher. 2000;18(5):192-8.

5. Baker PJ, Thompson R, Grogan A. Survival rates of cat-attacked birds admitted to RSPCA wildlife centres in the UK: Implications for cat owners and wildlife rehabilitators. Animal Welfare. 2018;27(4):305-18. doi: 10.7120/09627286.27.4.305.

6. Kelly A, Halstead C, Hunter D, Leighton K, Grogan A, Harris M. Factors affecting the likelihood of release of injured and orphaned woodpigeons (*Columba palumbus*). Animal Welfare. 2011;20(4):523-34.

7. Cousins RA, Battley PF, Gartrell BF. Impact injuries and probability of survival in a large semiurban endemic pigeon in New Zealand, *Hemiphaga novaeseelandiae*. Journal of Avian Medicine and Surgery. 2012;26(4):274.

8. Montesdeoca N, Calabuig P, Corbera JA, Cooper JE, Orós J. Causes of morbidity and mortality, and rehabilitation outcomes of birds in Gran Canaria Island, Spain. Bird Study. 2017;64(4):523-34. doi: 10.1080/00063657.2017.1411464.

9. De La Cruz SEW, Takekawa JY, Spragens KA, Yee J, Golightly RT, Massey G, et al. Post-release survival of surf scoters following an oil spill: An experimental approach to evaluating rehabilitation success. Marine Pollution Bulletin. 2013;67(1-2):100-6. doi: 10.1016/j.marpolbul.2012.11.027.

10. Duerr RS, Ziccardi MH, Gregory Massey J. Mortality during treatment: Factors affecting the survival of oiled, rehabilitated common murres (*Uria aalge*). Journal of Wildlife Diseases. 2016;52(3):495-505. doi: 10.7589/2015-03-054.

11. Naidoo V, Wolter K, Espie I, Kotze A. Vulture rescue and rehabilitation in South Africa: an urban perspective. Journal of the South African Veterinary Association. 2011;82(1):24-31. doi: 10.4102/jsava.v82i1.64.

12. Howard A, Hirschauer M, Monadjem A, Forbes N, Wolter K. Injuries, mortality rates, and release rates of endangered vultures admitted to a rehabilitation center in South Africa. Journal of Wildlife Rehabilitation. 2020;40(3):15-24.

13. Hernandez CL, Oster SC, Newbrey JL. Retrospective study of raptors treated at the Southeastern Raptor Center in Auburn, Alabama. Journal of Raptor Research. 2018;52(3):379-88. doi: 10.3356/jrr-17-16.1. PubMed PMID: WOS:000441062500011.

14. Komnenou AT, Georgopoulou I, Savvas I, Dessiris A. A retrospective study of presentation, treatment, and outcome of free-ranging raptors in Greece (1997–2000). Journal of Zoo and Wildlife Medicine. 2005;36(2):222-8.

15. Maphalala MI, Monadjem A, Bildstein KL, Hoffman B, Downs C. Causes of admission to a raptor rehabilitation centre and factors that can be used to predict the likelihood of release. African Journal of Ecology. 2021;00:1-8. doi: 10.1111/aje.12851.

16. Molina-López RA, Casal J, Darwich L. Final disposition and quality auditing of the rehabilitation process in wild raptors admitted to a wildlife rehabilitation centre in Catalonia, Spain, during a twelve year period (1995-2007). PLoS ONE. 2013;8(4):e60242. doi: 10.1371/journal.pone.0060242.

17. Montesdeoca N, Calabuig P, Corbera JA, Rocha J, Orós J. Final outcome of raptors admitted to the Tafira wildlife rehabilitation center, gran Canaria island, Spain (2003-2013). Animal Biodiversity and Conservation. 2017;40(2):211-20. doi: 10.32800/abc.2017.40.0211.

18. Rodríguez B, Rodríguez A, Siverio F, Siverio M. Causes of raptor admissions to a wildlife rehabilitation center in Tenerife (Canary Islands). Journal of Raptor Research. 2010;44(1):30-9. doi: 10.3356/JRR-09-40.1.

19. Sós-Koroknai V, Solymosi N, Kriko E, Toth T, Marosan M, Sos E. Examination of morbidity and the incidence of electrocution in common kestrels (*Falco tinnunculus*) admitted to the Wildlife Rescue Centre at the Budapest Zoo and Botanical Garden between 2014 and 2016. Magyar Allatorvosok Lapja. 2020;142(7):429-38. PubMed PMID: WOS:000560164500006.

20. Kelly A, Bland M. Admissions, diagnoses, and outcomes for Eurasian Sparrowhawks (*Accipiter nisus*) brought to a wildlife rehabilitation center in England. Journal of Raptor Research. 2006;40(3):231-5. doi: 10.3356/0892-1016(2006)40[231:Adaofe]2.0.Co;2. PubMed PMID: WOS:000241997700009.

21. Duke G, Redig P, Jones W. Recoveries and resightings of released rehabilitated raptors. Journal of Raptor Research. 1981;15(4):97-107.

22. Mariacher A, Gherardi R, Mastrorilli M, Melini D. Causes of admission and outcomes of long-eared owl (*Asio otus*) in wildlife rescue centres in Italy from 2010 to 2014. Avian Biology Research. 2016;9(4):282-6. doi: 10.3184/175815516X14739467542487.

23. Parsons NJ, Vanstreels RE, Schaefer AM. Prognostic indicators of rehabilitation outcomes for adult African penguins (*Spheniscus demersus*). Journal of Wildlife Diseases. 2018;54(1):54-65.

24. Barnett J, Westcott S. Distribution, demographics and survivorship of grey seal pups (*Halichoerus grypus*) rehabilitated in southwest England. Mammalia. 2001;65(3):349-61. doi: 10.1515/mamm.2001.65.3.349.

25. Martínez JC, Rosique AI, Royo MS. Causes of admission and final dispositions of hedgehogs admitted to three wildlife rehabilitation centers in eastern Spain. Hystrix. 2014;25(2):107-10. doi: 10.4404/hystrix-25.2-10248.

26. Santos ARBMF. Eastern cottontail rabbit (*Sylvilagus floridanus*) admission causes and corresponding outcomes at the Wildlife Rehabilitation Center of Minnesota: a retrospective study from 2011 to 2017 [PhD thesis]: Universidade de Lisboa, Faculdade de Medicina Veterinária; 2018.

27. Molony SE, Baker PJ, Garland L, Cuthill IC, Harris S. Factors that can be used to predict release rates for wildlife casualties. Animal Welfare. 2007;16(3):361-7.

28. Grogan A, Pulquério MJ, Cruz MJ, Oaten P, Thompson R, Grantham M, et al., editors. Factors affecting the welfare and rehabilitation of oiled murres (*Uria aalge*) in England and Wales, UK. The International Wildlife Rehabilitation Council symposium; 2011; Coral Springs, FL.

29. Altwegg R, Crawford RJM, Underhill LG, Williams AJ. Long-term survival of de-oiled Cape gannets *Morus capensis* after the Castillo de Bellver oil spill of 1983. Biological Conservation. 2008;141(7):1924-9. doi: 10.1016/j.biocon.2008.04.030.

30. Garcês A, Soeiro V, Lóio S, Sargo R, Sousa L, Silva F, et al. Outcomes, mortality causes, and pathological findings in european hedgehogs (*Erinaceus europeus*, Linnaeus 1758): A seventeen year retrospective analysis in the North of Portugal. Animals. 2020;10(8):1-13. doi: 10.3390/ani10081305.

31. Serangeli M, Cistrone L, Ancillotto L, Tomassini A, Russo D. The post-release fate of hand-reared orphaned bats: survival and habitat selection. Animal Welfare-The UFAW Journal. 2012;21(1):9.

32. Saran K, Parker G, Parker R, Dickman C. Rehabilitation as a conservation tool: a case study using the common wombat. Pacific Conservation Biology. 2011;17(4):310-9.

33. Griffith JE, Higgins DP. Diagnosis, treatment and outcomes for koala chlamydiosis at a rehabilitation facility (1995-2005). Australian Veterinary Journal. 2012;90(11):457-63. doi: 10.1111/j.1751-0813.2012.00963.x.

34. Camphuysen C, Duiven P, Harris M, Leopold M. Recoveries of guillemots ringed in the Netherlands: the survival of rehabilitated oiled seabirds. SULA. 1997;11(3):157-74.

35. Greig DJ, Gulland FMD, Rios CA, Hall AJ. Hematology and serum chemistry in stranded and wildcaught harbor seals in central California: Reference intervals, predictors of survival, and parameters affecting blood variables. Journal of Wildlife Diseases. 2010;46(4):1172-84. doi: 10.7589/0090-3558-46.4.1172.

36. Charalambous R, Narayan E. A 29-year retrospective analysis of koala rescues in New South Wales, Australia. Plos One. 2020;15(10):e0239182. doi: 10.1371/journal.pone.0239182. PubMed PMID: WOS:000588374000051.

37. Anderson DW, Gress F, Fry DM. Survival and dispersal of oiled brown pelicans after rehabilitation and release. Marine Pollution Bulletin. 1996;32(10):711-8. doi: <https://doi.org/10.1016/0025-326X(96)00027-6>.

38. Tribe A, editor Measuring the success of wildlife rehabilitation. National Wildlife Rehabilitation Conference, Surfers Paradise; 2005; Gold Coast.

39. Kelly A, Scrivens R, Grogan A. Post-release survival of orphaned wild-born polecats *Mustela putorius* reared in captivity at a wildlife rehabilitation centre in England. Endangered Species Research. 2010;12(2):107-15. doi: 10.3354/esr00299.

40. Haynes E, Erb HN, Nevis J. Statistical analysis of juvenile American robin rehabilitation at Willowbrook Wildlife Center, Illinois, USA: can admission weight be used to predict rehabilitation outcome? Journal of Wildlife Rehabilitation. 2013;33(1):19-23.

41. MacRae A, Haulena M, Fraser D. The effect of diet and feeding level on survival and weight gain of hand‐raised harbor seal pups (*Phoca vitulina*). Zoo Biology. 2011;30(5):532-41.

42. Drake A. Mallard duckling care and survival at a wildlife rehabilitation center [PhD thesis]: University of British Columbia; 2007.

43. Herbert C, Gillies C, Mella V, Webster K, Keong J, Jennings A, et al., editors. Brushtail Possums in Care: Factors influencing post-release survival and the potential impacts of stress on release outcomes. Australian WIldlife Rehabilitation Conference; 2018; Sydney.

44. Rogers DI, Battley PF, Sparrow J, Koolhaas A, Hassell CJ. Treatment of capture myopathy in shorebirds: a successful trial in northwestern Australia. Journal of Field Ornithology. 2004;75(2):157-64. doi: 10.1648/0273-8570-75.2.157. PubMed PMID: WOS:000220930400007.

45. Weston MA, Dann P, Jessop R, Fallaw J, Dakin R, Ball D. Can oiled shorebirds and their nests and eggs be successfully rehabilitated? A case study involving the threatened hooded plover *Thinornis rubricollis* in south-eastern Australia. Waterbirds. 2008;31(1):127-32. doi: 10.1675/1524-4695(2008)31[127:COSATN]2.0.CO;2.

46. Golightly RT, Newman SH, Craig EN, Carter HR, Mazet JAK. Survival and behavior of western gulls following exposure to oil and rehabilitation. Wildlife Society Bulletin. 2002;30(2):539-46.

47. Parsons N, Underhill L. Oiled and injured African penguins *Spheniscus demersus* and other seabirds admitted for rehabilitation in the Western Cape, South Africa, 2001 and 2002. African Journal of Marine Science. 2005;27(1):289-96.

48. Thompson LJ, Hoffman B, Brown M. Causes of admissions to a raptor rehabilitation centre in KwaZulu-Natal, South Africa. African Zoology. 2013;48(2):359-66.

49. Newman SH, Golightly RT, Craig EN, Carter HR, Kreuder C. The effects of petroleum exposure and rehabilitation on post-release survival, behavior, and blood health indices: A common murre (*Uria aalge*) case study following the Stuyvesant petroleum spill. Final Report. UC Davis, CA: 2004.

50. Burton E, Tribe A. The rescue and rehabilitation of koalas (*Phascolarctos cinereus*) in southeast Queensland. Animals. 2016;6(9):56. doi: 10.3390/ani6090056. PubMed PMID: WOS:000422958400006.

51. Paul G, Friend DG. Comparison of outcomes using two milk replacer formulas based on commercially available products in two species of infant cottontail rabbits. Journal of Wildlife Rehabilitation. 2017;37(1):13-9.

52. Yarnell RW, Surgey J, Grogan A, Thompson R, Davies K, Kimbrough C, et al. Should rehabilitated hedgehogs be released in winter? A comparison of survival, nest use and weight change in wild and rescued animals. European Journal of Wildlife Research. 2019;65(1):6. doi: 10.1007/s10344-018-1244-4.

53. Molony SE, Dowding CV, Baker PJ, Cuthill IC, Harris S. The effect of translocation and temporary captivity on wildlife rehabilitation success: An experimental study using European hedgehogs (*Erinaceus europaeus*). Biological Conservation. 2006;130(4):530-7. doi: 10.1016/j.biocon.2006.01.015.

54. Beecham JJ, De Gabriel Hernando M, Karamanlidis AA, Beausoleil RA, Burguess K, Jeong D-H, et al. Management implications for releasing orphaned, captive-reared bears back to the wild. The Journal of Wildlife Management. 2015;79(8):1327-36. doi: <https://doi.org/10.1002/jwmg.941>.

55. Hashem BJ. Evaluating the success of an orphaned American black bear (*Ursus americanus*) rehabilitation program in Virginia. Journal of Wildlife Rehabilitation. 2019;39(2):7-12.

56. Kido N, Omiya T, Kamegaya C, Wada Y, Takahashi M, Yamamoto Y. Effective treatment for improving the survival rate of raccoon dogs infected with *Sarcoptes scabiei*. Journal of Veterinary Medical Science. 2014;76(8):1169-72.

57. Goldingay RL, Dobner B. Home range areas of koalas in an urban area of north-east New South Wales. Australian Mammalogy. 2014;36(1):74-80. doi: 10.1071/am12049. PubMed PMID: WOS:000332781400010.

58. Underhill LG, Bartlett PA, Baumann L, Crawford RJ, Dyer BM, Gildenhuys A, et al. Mortality and survival of African penguins *Spheniscus demersus* involved in the Apollo Sea oil spill: an evaluation of rehabilitation efforts. Ibis. 1999;141(1):29-37.

59. Wernham C, Peach WJ, Browne SJ. Survival rates of rehabilitated guillemots. BTO Research Report No. 186 Thetford, Norfolk: British Trust for Ornithology, 1997 0903793717.

60. Beringer J, Mabry P, Meyer T, Wallendorf M, Eddleman WR. Post-release survival of rehabilitated white-tailed deer fawns in Missouri. Wildlife Society Bulletin. 2004;32(3):732-8. doi: 10.2193/0091-7648(2004)032[0732:PSORWD]2.0.CO;2.

61. Houser A, Gusset M, Bragg CJ, Boast LK, Somers MJ. Pre-release hunting training and post-release monitoring are key components in the rehabilitation of orphaned large felids. African Journal of Wildlife Research. 2011;41(1):11-20. doi: 10.3957/056.041.0111.

62. Miazga K, Joubert J, Sinclair M, Cywińska A. Releasing three orphaned white rhinoceroses (*Ceratotherium simum*) to the game reserve in South Africa. Rehabilitation, translocation and post-release observations. Animals. 2020;10(12):1-15. doi: 10.3390/ani10122224.

63. Nicholson TE, Mayer KA, Staedler MM, Johnson AB. Effects of rearing methods on survival of released free-ranging juvenile southern sea otters. Biological Conservation. 2007;138(3):313-20. doi: <https://doi.org/10.1016/j.biocon.2007.04.026>.

64. Williams SC, Gregonis MA. Survival and movement of rehabilitated white‐tailed deer fawns in Connecticut. Wildlife Society Bulletin. 2015;39(3):664-9.

65. Houser A. Spoor density, movement and rehabilitation of cheetahs in Botswana [Masters thesis]: University of Pretoria; 2009.

66. Suarez C, Gamboa E, Claver P, Nassar-Montoya F. Survival and adaptation of a released group of confiscated capuchin monkeys. Animal Welfare. 2001;10(2):191-203.

67. Kelly A, Goodwin S, Grogan A, Mathews F. Further evidence for post-release survival of hand-reared, orphaned bats based on radio-tracking and ring-return data. Animal Welfare-The UFAW Journal. 2012;21(1):27.

68. Groom CJ, Warren K, Mawson PR. Survival and reintegration of rehabilitated Carnaby's cockatoos *Zanda latirostris* into wild flocks. Bird Conservation International. 2018;28(1):86.

69. Wimberger K, Downs C, Perin M. Postrelease success of two rehabilitated vervet monkey (*Chlorocebus aethiops*) troops in KwaZulu-Natal, South Africa. Folia Primatologica. 2010;81:96–108.

70. Blair CD, Muller LI, Clark JD, Stiver WH. Survival and conflict behavior of American black bears after rehabilitation. Journal of Wildlife Management. 2020;84(1):75-84. doi: 10.1002/jwmg.21783.

71. Meyer FC. Survival and distribution of Temminck’s pangolin (*Smutsia temminckii*) retrieved from the illegal wildlife trade in South Africa [Masters thesis]: University of Venda; 2020.

72. Ashraf N, Dadda T, Boro P, Akhtar N. Walking the Bears - Rehabilitation of Asiatic black bears in Arunachal Pradesh. New Delhi: Wildlife Trust of India; 2008. 1-125 p.

73. Campbell L, Croft D, editors. Comparison of hard and soft release of hand reared eastern grey kangaroos. Veterinary conservation biology, wildlife health and management in Australasia, proceedings of international joint conference; 2001; Sydney: Taronga Zoo.

74. Barman R, Choudhury B, Ashraf NVK, Menon V. Rehabilitation of greater one-horned rhinoceros calves in Manas National Park, a World Heritage Site in India. Journal of Wildlife Rehabilitation. 2019;39(1):17-26.

75. Morris PA. Released, rehabilitated hedgehogs: A follow-up study in Jersey. Animal Welfare. 1997;6(4):317-27.

76. Morris P, Meakin K, Sharafi S. The behaviour and survival of rehabilitated hedgehogs (*Erinaceus europaeus*). Animal Welfare. 1993;2(1):53-66.

77. Augee ML, Smith B, Rose S. Survival of wild and hand-reared ringtail possums (*Pseudocheirus peregrinus*) in bushland near Sydney. Wildlife Research. 1996;23(1):99-108. doi: 10.1071/WR9960099.

78. Neyens J, Hirst S, editors. Survival of released rehabilitated northern brush tailed possums (*Trichosurus arnhemensis*). Australian Wildlife Rehabilitation Conference; 2014; Hobart.

79. Smith WE, Pekins PJ, Timmins AA, Kilham B. Short-term fate of rehabilitated orphan black bears released in New Hampshire. Human–Wildlife Interactions. 2016;10(2):14.

80. Russell B, Smith B, Augee M. Changes to a population of common ringtail possums (*Pseudocheirus peregrinus*) after bushfire. Wildlife Research. 2003;30(4):389-96.

81. Hamilton LL, Zwank PJ, Olsen GH. Movements and survival of released rehabilitated hawks. Raptor Research. 1988;22(1):22-6.

82. Guy AJ, Stone OML, Curnoe D. Assessment of the release of rehabilitated vervet monkeys into the Ntendeka Wilderness Area, KwaZulu-Natal, South Africa: a case study. Primates. 2012;53(2):171-9. doi: 10.1007/s10329-011-0292-0. PubMed PMID: WOS:000302572100007.

83. Guy AJ. Release of rehabilitated *Chlorocebus aethiops* to Isishlengeni Game Farm in KwaZulu-Natal, South Africa. Journal for Nature Conservation. 2013;21(4):214-6. doi: 10.1016/j.jnc.2013.01.002.

84. Martell M, Redig P, Nibe J, Buhl G, Frenzel D. Survival and movements of released rehabilitated bald eagles. Biological Conservation. 1992;62(3):231. doi: 10.1016/0006-3207(92)91069-5.

85. Lunney D, Gresser SM, Mahon PS, Matthews A. Post-fire survival and reproduction of rehabilitated and unburnt koalas. Biological Conservation. 2004;120(4):567-75. doi: 10.1016/j.biocon.2004.03.029. PubMed PMID: WOS:000224014100012.

86. Ridgeway P, editor Rewilding ecosystems through wildlife rehabilitation – a successful trial in Western Sydney. Australian Wildlife Rehabilitation Conference; 2018; Sydney.

87. Narayan E, Vanderneut T. Physiological stress in rescued wild koalas are influenced by habitat demographics, environmental stressors, and clinical intervention. Frontiers in Endocrinology. 2019;10:18. doi: 10.3389/fendo.2019.00018. PubMed PMID: WOS:000457063500002.
